# *NUSAP1* and *PCLAF* (*KIA0101*) Downregulation by Neoadjuvant Therapy is Associated with Better Therapeutic Outcomes and Survival in Breast Cancer

**DOI:** 10.1155/2022/6001947

**Published:** 2022-11-28

**Authors:** Gerardo I. Magallanes-Garza, Sandra K. Santuario-Facio, Saúl Lira-Albarrán, Arlina F. Varela-Varela, Servando Cardona-Huerta, Pablo Ruiz-Flores, Jorge Haro-Santa-Cruz, Yadira X. Perez-Paramo, Gabriela Sofia Gomez-Macias, Daniel Davila-Gonzalez, Javier Valero-Gomez, Gissela Borrego-Soto, Augusto Rojas-Martinez, Rocio Ortiz-Lopez

**Affiliations:** ^1^Tecnologico de Monterrey, Escuela de Medicina y Ciencias de La Salud, Monterrey, Mexico; ^2^Tecnologico de Monterrey, Servicio de Ginecologia y Obstetricia, Tec Salud, Monterrey, Mexico; ^3^Tecnologico de Monterrey, Centro de Cancer de Mama, Hospital Zambrano Hellion, San Pedro Garza Garcia, Mexico; ^4^Tecnologico de Monterrey, The Institute for Obesity Research, Monterrey, Mexico; ^5^Universidad Autonoma de Coahuila, Centro de Investigacion Biomedica, Facultad de Medicina, Unidad Torreon, Torreon, Mexico; ^6^Tecnologico de Monterrey, Direccion Nacional de Investigacion Clinica TecSalud, Monterrey, Mexico; ^7^Instituto Mexicano Del Seguro Social, Unidad Medica de Alta Especialidad No. 25, Monterrey, Mexico; ^8^Washington State University, Pharmaceutical Sciences Department, College of Pharmacy Spokane, WA, USA; ^9^Universidad Autonoma de Nuevo Leon, Facultad de Medicina, Monterrey, Mexico; ^10^University of Texas at Austin, Department of Molecular Biosciences, Austin, TX, USA; ^11^Universidad Autonoma de Nuevo Leon, CIDICS, Monterrey, Mexico

## Abstract

**Purpose:**

To evaluate whether changes in genomic expression that occur beginning with breast cancer (BC) diagnosis and through to tumor resection after neoadjuvant chemotherapy (NCT) reveal biomarkers that can help predict therapeutic response and survival.

**Materials and Methods:**

We determined gene expression profiles based on microarrays in tumor samples from 39 BC patients who showed pathologic complete response (pCR) or therapeutic failure (non-pCR) after NCT (cyclophosphamide-doxorubicin/epirubicin). Based on unsupervised clustering of gene expression, together with functional enrichment analyses of differentially expressed genes, we selected *NUSAP1*, *PCLAF*, *MME*, and *DST*. We evaluated the NCT response and the expression of these four genes in BC histologic subtypes. In addition, we study the presence of tumor-infiltrating lymphocytes. Finally, we analyze the correlation between *NUSAP1* and *PCLAF* against disease-free survival (DFS) and overall survival (OS).

**Results:**

A signature of 43 differentially expressed genes discriminated pCR from non-pCR patients (|fold change >2|, false discovery rate <0.05) only in biopsies taken after surgery. Patients achieving pCR showed downregulation of *NUSAP1* and *PCLAF* in tumor tissues and increased DFS and OS, while overexpression of these genes correlated with poor therapeutic response and OS. These genes are involved in the regulation of mitotic division.

**Conclusions:**

The downregulation of *NUSAP1* and *PCLAF* after NCT is associated with the tumor response to chemotherapy and patient survival.

## 1. Introduction

Therapeutic response and prognosis in breast cancer (BC) are affected by such factors as patient age [1], clinical stage [2], tumor histopathology, and molecular subtypes [3]. Gene expression signatures performed before therapy can provide additional information on tumor biology, and algorithms have been developed to predict the risk of relapse and survival and define the best treatment options [4–6]. A program of genomic testing may allow for identifying low-risk tumors associated with a favorable prognosis. Also, it would facilitate therapeutic decision-making for aggressive tumors that respond poorly to conventional therapies. In this regard, transcriptional signatures can identify gene expression patterns related to chemotherapy resistance, immune system response, and tumor invasion [7–10].

Comparisons of gene expression analyses of biopsy specimens taken before and after neoadjuvant chemotherapy treatment (NCT) may help define tumor molecular adaptations to a specific chemotherapeutic agent or regime [7–10]. The pathologic complete response (pCR) in BC is defined as the absence of all invasive tumor tissue in the breast and axillary lymph nodes after the completion of NCT cycles [11, 12]. The achievement of pCR after NCT correlates with patient survival [12]. Alternative treatment regimens may improve survival when pCR is not achieved [13]. Comparisons of the changing patterns of transcriptional signatures in response to chemotherapy may enable predictions of clinical response and prognosis and, sometimes, recognize new response biomarkers of specific canonical pathways related to treatment resistance and recurrence.

No genomic signature defines therapeutic alternatives in patients with incomplete pathologic response (non-pCR). Therefore, the identification of gene expression profiles in tumor tissue after NCT that are associated with a good or an inadequate pathological response or with survival could facilitate the identification of patients who could benefit from second-line adjuvant treatment or improve clinical follow-up, as has been shown in some studies assessing pathologic response [14]. In addition, a review of the canonical pathways in which these genes are involved could also provide potential therapeutic targets or identify markers for high-risk patients who require closer follow-up.

This work aimed to analyze changes in genomic expression in primary BC tumors in patients undergoing NCT and to identify genes associated with prognosis in nonresponding patients. These potential biomarkers could guide new pharmaceutical interventions for the second line of treatment. After validation, our studies showed that downregulation of *NUSAP1* and *PCLAF* (Previous Symbol HGNC: *KIAA0101*) and overexpression of *MME* and *DST* in tumor biopsies of patients significantly correlated with pCR after NCT disease-free survival (DFS) and overall survival (OS). *NUSAP1* is involved in cell proliferation and migration, and *PCLAF* participates in cell cycle control and apoptosis [15, 16]. Overexpression of these genes has each been correlated with tumor progression and metastasis [17, 18]. Downregulation of *MME* is associated with tumor recurrence and metastasis [19]. Underexpression of *DST*, which produces a cytoskeletal protein, promotes breast cancer progression independently of tumor hormonal status [20].

## 2. Materials and Methods

### 2.1. Patient Population

Patients with BC were enrolled in the study at the Centro de Cancer de Mama (Breast Cancer Center) Hospital San Jose TecSalud in Monterrey, Mexico. The Institutional Review Board of the School of Medicine of Tecnologico de Monterrey (CONBIOETICA 19 CEI 011-2016-10-17) authorized the research protocol with the number: P000088-Altru-Pro-CI-CR002. Following the Declaration of Helsinki, informed written consent was obtained from all patients participating in this study. Tissue samples were collected from 54 patients with clinical and or radiologic diagnoses of BC (tumor size >2 cm and palpable lymph nodes) from July 2011 to October 2014.

### 2.2. Neoadjuvant Chemotherapeutic Regimens

Regimens were established according to the clinical stage and the immunohistochemistry of the breast tumors by medical oncologists. They consisted of 4 cycles every three weeks of either intravenous cyclophosphamide (500–1500 mg/m^2^) and doxorubicin (≥40 mg/m^2^) or intravenous cyclophosphamide (500–1500 mg/m^2^) and epirubicin (≥60 mg/m^2^). After receiving either of these regimens, patients received 12 weekly cycles of intravenous paclitaxel (80 mg/m^2^) administered over 1 hr [21]. In patients who demonstrated drug toxicity, cycles of carboplatin replaced the drug responsible for the toxicity [22]. Subsequently, surgical resection of the breast was performed on each patient. Some patients received selected adjuvant therapy after NCT (46% tamoxifen and 15% trastuzumab), as recommended by the attending oncologist. In such cases, the chemotherapeutic drug was chosen according to individual patient characteristics and clinical guidelines (e.g., trastuzumab and tamoxifen).

### 2.3. Tumor Sample Collection

Two tissue samples were collected from each patient: a biopsy sample (BS) before NCT and a surgery sample (SS) collected after completing the cycles of NCT. Thick needle puncture biopsies were obtained using a Bard Magnum 12 Fr gauge needle. Tumor location was marked at diagnosis using the carbon tracking technique [23]. Six to eight tissue cylinders were obtained from each patient. Four samples were used for histopathologic analysis, and three pieces were preserved in RNAlater solution (Sigma-Aldrich; Burlington, MA) for genomic analysis. The SS were obtained from surgeries for local-regional control (modified radical mastectomy in most cases). Tissues were sent to pathology for histopathologic and immunohistochemistry analysis. In addition, a 2 × 1 cm piece, marked by the carbon track used during the diagnostic biopsy procedure, was preserved in RNAlater solution for the gene expression analysis.

### 2.4. Immunohistochemistry Analysis and Assessment of Tumor-Infiltrating Lymphocytes in BC Samples

Samples were obtained from each patient for hematoxylin-eosin staining and immunohistochemistry for estrogen receptor (ER), progesterone receptor (PR), and HER2/neu. The histologic grade of the core needle biopsies was obtained before neoadjuvant therapy using the Bloom-Richardson scores [24]. The stage of breast cancer was determined according to the American Joint Committee on Cancer [25]. The percentage of tumor-infiltrating lymphocytes (TILs) was assessed following the International TILs Working Group 2014 in breast cancer [26]. A complete methodology for TIL assessment has been previously described [27]. Immunohistochemistry for CD3^+^, CD4^+^, and CD8^+^ was also performed on the core needle biopsies before NCT to define lymphocyte immunophenotypes, following the American Society of Clinical Oncology/College of American Pathologists guidelines [28].

### 2.5. Treatment Response

One pathologist evaluated surgical specimens and assessed tumor response to NCT using the Miller–Payne grading system. For this study, a Miller-Payne grade 5 score was pCR, and the remaining scores (including partial pathologic response) were classified as non-pCR [29].

### 2.6. RNA Isolation and Microarray Hybridization

RNA isolation from BS and SS was prepared using RNeasy Fibrous Tissue Mini Kit (Qiagen; Germantown, MA) following the manufacturer's instructions. RNA quality was assessed by capillary electrophoresis using the Experion Automated Electrophoresis Station (Bio-Rad; Hercules, CA). Processing and microarray hybridization from the selected RNA samples were conducted using the GeneChip 3' IVT Express Kit (Thermo Fisher Scientific; Waltham, MA) and GeneChip Human Genome U133 Plus 2.0 Array (Applied Biosystems; Santa Clara, CA), according to manufacturer's instructions and as previously described [30, 31].

### 2.7. Microarray Data Processing

Normalization was performed using robust multiarray average (RMA) [32]. Probes with a mean expression <3 (logarithmic scale derived from RMA) were also removed from the further analysis. The differential gene expression analysis was performed using a *t*-test with multiple comparison corrections using the false discovery rate (FDR) method [33]. We considered the probes positive with an FDR <0.05. The differentially expressed genes (DEGs) were those with |fold change (FC) > 2| and FDR <0.05 in every contrast evaluated. These analyses were completed using the free Applied Biosystems Transcriptome Analysis Console (TAC) 4.0.1 software (Thermo Fisher Scientific).

### 2.8. Functional Enrichment Analysis

The functional enrichment analysis was performed using g:Profiler *β* (version e106_e53_p16_12c39de) with the g:SCS multiple testing correction methods, applying a significance threshold of 0.05 [34] and uploading the list of DEGs from every contrast evaluated. The nomenclature of molecular functions, biological processes, and cellular components used the terms of the Gene Ontology Consortium [35]. In addition, the enriched canonical pathways were identified using KEGG [36], Reactome [37], and WikiPathways [38].

### 2.9. Ingenuity Pathway Analysis

The core analysis generated with QIAGEN IPA (QIAGEN Inc., https://digitalinsights.qiagen.com/IPA, accessed on 17 September 2022) identified the enriched bio-functions and canonical pathways (*p* − value < 0.01 using the right-tailed Fisher´s exact test) defined by the Ingenuity Knowledge Base as well as the networks with the highest number of molecules involved [39] using the list of DEGs. Additionally, based on a hypothesis-driven approach to DEGs´ effect on the regulation of mitosis, a molecule activity predictor analysis (MAP) by IPA was done.

### 2.10. Real-Time qPCR Validation

To validate microarray data, we selected four genes based on the microarray differential gene expression results and the functional enrichment analysis with g:Profiler *β* and IPA: two overexpressed genes (*MME* and *DST*) and two underexpressed genes (*NUSAP1* and *PCLAF*) in SS tissues. In addition, *GRAMD1A* was used as an endogenous gene control due to a low variation between samples [30]. Expression analyses were assessed using predesigned hydrolysis probes (*MME*, Hs00153510_m1; *DST*, Hs00156137_m1; *NUSAP1*, Hs01006195_m1; *PCLAF*, Hs00207134_m1; *GRAMD1A*, Hs.PT.5840681431) (Thermo Fisher Scientific and IDT for *GRAMD1A*). Total RNA aliquots used for microarray assays were analyzed through qPCR using the Applied Biosystems QuantStudio 3 Real-Time PCR System (Thermo Fisher). Cycle threshold (*Ct*) means for each gene were used to calculate Δ*Ct* (problem minus endogenous), and 2^−ΔCt^ analysis was done using calculated Δ*Ct* for all genes. The gene expression of pCR and non-pCR groups was compared based on the relative expression 2^−ΔCt^ evaluated from qPCR data from all genes after normalization with *GRAMD1A*. An unpaired *t-*test with Welch's correction was used to establish differences (*p* − value < 0.05).

### 2.11. Evaluation of the Differences in Disease-free Survival and Overall Survival

The SS gene expression values with DFS and OS were evaluated in 39 patients. In addition, the differences in OS were assessed based on a log-rank (Mantel-Cox) test that compares Kaplan–Meier survival curves [GraphPad Prism Windows version 6.01 (La Jolla, CA)]. A *p* − value < 0.05 was statistically significant.

For external validation, Kaplan–Meier Plotter (https://kmplot.com/analysis/) online database [40, 41] was used to analyze the OS correlated to high vs. low gene mRNA expression levels. The Kaplan–Meier Plotter split the BC patient (*n* = 1402) samples into two groups according to their median mRNA levels. The Affymetrix probe IDs used for the Kaplan–Meier analysis were *PCLAF* 202503_s_at and *NUSAP1* 219978_s_at.

## 3. Results

### 3.1. Patients

Fifty-four patients were enrolled in the study, but only 44 paired (BS and SS) samples satisfied the RNA quality and quantity standards needed for the microarray analysis. In addition, five samples were eliminated because they failed to achieve quality standards after microarray hybridization, leaving 39 patient sample sizes for the final analyses. The clinical characteristics of the patients are described in Table 1. According to the Miller–Payne grading system, only 8 (20.5%) of the 39 patients reached pCR.

### 3.2. Gene Expression Profile Analysis

**Figure 1:**
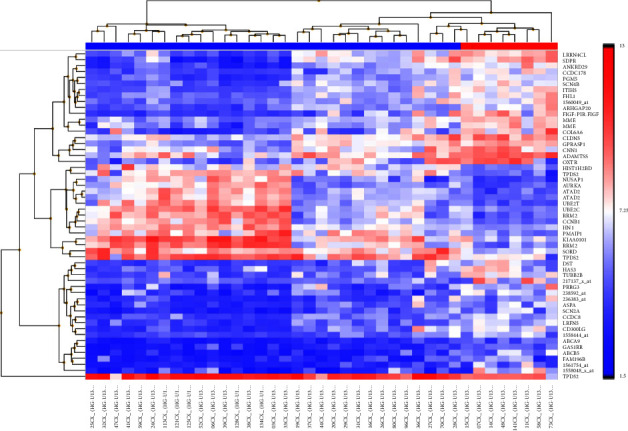
Unsupervised clustering of gene expression data in SS samples: pCR (*n* = 8) vs. non-pCR (*n* = 31). In the top row, pCR samples are denoted by the red header, and the blue title indicates non-pCR samples. The heatmap shows one sample for each column and one gene or probe for each horizontal line. The color indicates gene expression value intensities, where the gradient pink-red represents overexpression, and the gradient light blue-dark blue represents underexpression.

The following comparisons were made between SS and BS microarray data in pCR and non-pCR patients to assess the gene expression modifications induced by NCT: pCR-SS vs. pCR-BS (Supplementary Figure S1), non-pCR-SS vs. non-pCR-BS (Supplementary Figure S2), pCR-BS vs. and non-pCR-BS, and pCR-SS vs. non-pCR-SS (Figure 1).

### 3.3. Functional Enrichment Analysis

The first comparison pCR-SS vs. pCR-BS identified fourteen differentially expressed genes (|FC > 2|, FDR <0.05, DEGs) (Supplementary File S1a). The overrepresentation analysis using this list of DEGs (Supplementary File S1b) included the molecular functions (MFs): DNA-binding transcription activator activity, RNA polymerase II-specific and the protein tyrosine/serine/threonine phosphatase activity. Interestingly, the identified enriched canonical pathway Nuclear Events (kinase and transcription factor activation) integrates the MFs overrepresented and highlight the effects of kinase and phosphatase activity on transcription factors. The second contrast non-pCR-SS vs. non-pCR-BS identified only four DEGs (Supplementary File S2a), and the unique MF overrepresented was nicotinamide phosphoribosyltransferase activity (Supplementary File S2b). No DEGs were identified in the third comparison pCR-BS vs. non-pCR-BS. The most interesting contrast was pCR-SS vs. non-pCR-SS, with a transcriptional signature of 43 DEGs (Supplementary File S3a). The overrepresented biological process: regulation of the mitotic cell cycle (Supplementary File S3b) suggests that this contrast could help us identify potential biomarkers associated with the clinical outcomes of breast cancer. So, we evaluated it by ingenuity pathway analysis (IPA).

### 3.4. Ingenuity Pathway Analysis

**Figure 2:**
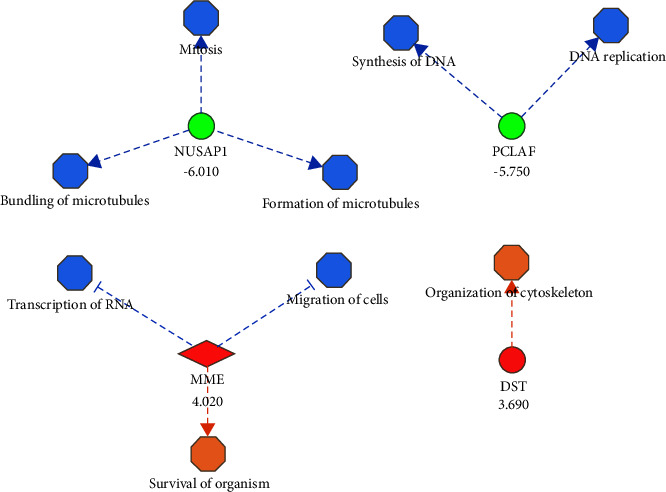
Molecule activity prediction of bio-functions associated with breast cancer using differentially expressed genes identified in the contrast pCR vs. non-pCR from SS samples. Colors indicated the predicted relationship between gene expression levels and bio-functions: green: down-regulated genes; red: up-regulated genes. Blue: bio-function inhibited; orange: bio-function activated. The blue line leads to inhibition; the orange line leads to activation. The fold change of every differentially expressed gene appears below from its gene symbol.

**Figure 3:**
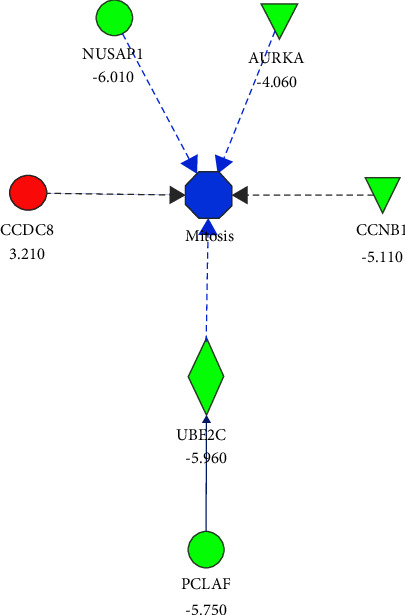
Molecule activity prediction of the mitosis biofunction using differentially expressed genes identified in the contrast pCR vs. non-pCR from SS samples. Colors indicated the predicted relationship between gene expression levels and biofunctions. Green: down-regulated genes; red: up-regulated genes. Blue: bio-function inhibited. The blue line leads to inhibition; the gray line indicates an effect not predicted. The fold change of every differentially expressed gene appears below from its gene symbol.

The core analysis by IPA using the list of 43 differentially expressed genes in the contrast pCR-SS vs. non-pCR-SS identified breast cancer as an enriched bio-function (Supplementary File S3c). In this regard, the most relevant enriched canonical pathways were cell Cycle: G2/M DNA damage checkpoint regulation, breast cancer regulation by Stathmin1, and molecular mechanisms of cancer (Supplementary File S3d). Interestingly, the MAP analysis based on the differential expression of some essential genes in the same contrast predicted inhibited bio-functions involved in breast cancer progression (Figure 2). For instance, the downregulation of *NUSAP1* and *PCLAF* inhibited mitosis and synthesis of DNA, respectively. The upregulation of *MME* inhibited the migration of cells, and the upregulation of *DST* activated the organization of the cytoskeleton. Furthermore, the MAP of mitosis bio-function predicted it as inhibited because of the downregulation of *NUSAP1* and *AURKA* that directly modified this critical process in cancer development. Indeed, *PCLAF* also influences mitosis by inhibiting *UBE2C* (Figure 3). In summary, the functional enrichment analysis identified *NUSAP1*, *PCLAF*, *MME*, and *DST* as potential biomarkers to be validated by RT-qPCR and evaluated in survival analyses.

### 3.5. RT-qPCR Validation

Four genes (*DST*, *MME*, *NUSAP1*, and *PCLAF*) were selected to validate the microarrays by RT-qPCR. Unfortunately, the remaining DNA from the tissue sample was scarce; therefore, only 31 (pCR = 5, non-pCR = 26) of 39 samples had enough quality and quantity of total RNA to perform this analysis. Supplementary Figure S3 shows the box plot of *NUSAP1* and *PCLAF* (Figures S3a and S3b, respectively) and *DST* and *MME* expression (Figures S3c and S3d, respectively). This analysis confirmed the expression patterns of these DEGs in the microarray.

### 3.6. *NUSAP1* and *PCLAF* Gene Expression

**Figure 4:**
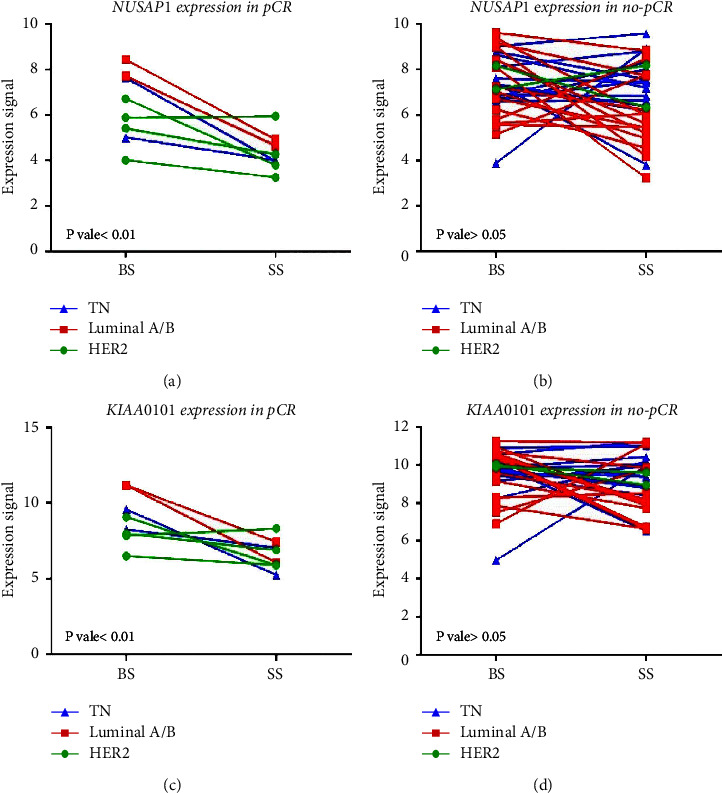
*NUSAP1* and *PCLAF/KIAA0101* gene expression based on microarray data. (a) *NUSAP1 gene expression in BS and SS in pCR* and (b) non-pCR patients, respectively. (c) PCLAF (Previous symbol KIAA0101) gene expression in BS and SS in pCR and (d) non-pCR (d) patients, respectively. Blue lines and triangles, triple-negative molecular subtype; red lines and squares, luminal A/B molecular subtype; green lines and circles, HER2 molecular subtypes. Two-way ANOVA was performed, and a *p* − value < 0.05 was considered significant.

In the subset of patients achieving pCR, *NUSAP1* and *PCLAF* gene expressions were higher in the BS than in the SS samples (two-way ANOVA, *F* = 22.12, *p* − value = 0.0053) (Figures 4(a) and 4(c). In contrast, there was no significant difference in the expression values in the non-pCR groups (two-way ANOVA, *F* = 1.246, *p* − value = 0.27) (Figures 4(b) and 4(d)). It is important to note that the expression of *NUSAP1* after NCT was significantly higher in luminal B tumors than in the rest of the histologic subtypes (F test = 4.88, *p* − value = 0.006) (Supplementary Figure S4).

### 3.7. Expression of *NUSAP1* and *PCLAF1* and Response to Treatment

tResponse to NCT was considered the primary response variable and was evaluated using the Miller–Payne grading system. The association between the *NUSAP1* and *PCLAF* genes with response to treatment was tested. The expression of *NUSAP1* and *PCLAF* after NCT were inversely associated with pCR, implying that the downregulation of these genes had a favorable effect on the patient, as shown in Table 2 (*NUSAP1*: OR = 0.00, CI95% = 0.00–0.99, *p* = 0.0417; *PCLAF*: OR = 0.01, CI 95% = 0.0009–0.1383, *p* = 0.0001).

### 3.8. Tumor-Infiltrating Lymphocytes (TILs) in BC Samples

A correlation between TILs and gene expression levels of *NUSAP1* or *PCLAF* before NCT was not observed (*r* = 0.10, *p* = 0.65, 95% CI −0.39–0.25) or *PCLAF* (*r* = −0.07, *p* = 0.54, 95% CI −0.22–0.40). Representative images of TILs evaluation are shown in Supplementary Figure S5.

### 3.9. Disease-free Survival and Overall Survival

**Figure 5:**
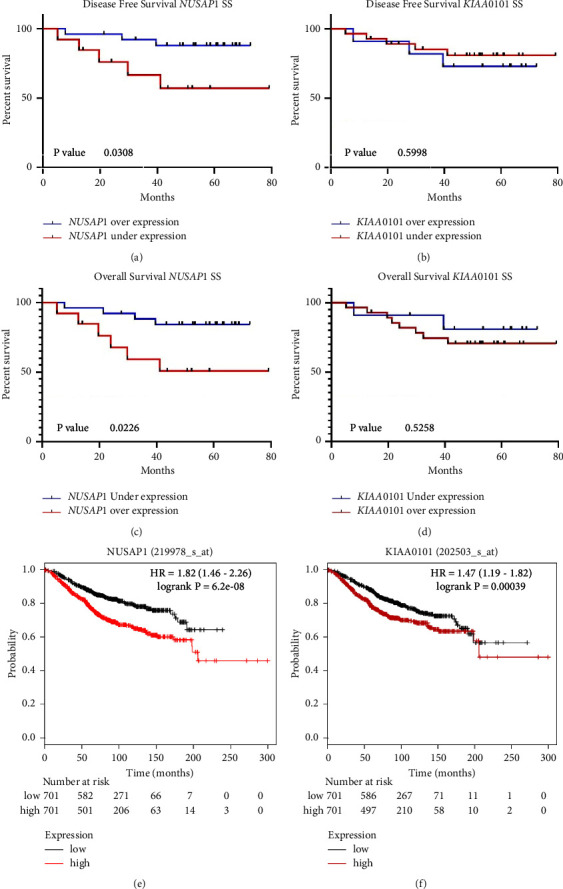
Disease-free survival and overall survival against *NUSAP1* and *PCLAF*/*KIAA0101* gene expression profiles in surgical samples (SS). (a-b) DFS curves considering *NUSAP1* and *PCLAF*/*KIAA0101* gene expression profiles after NCT (SS), respectively. Blue lines, underexpression; red lines, overexpression. (c-d) OS curves considering *NUSAP1* and *PCLAF*/*KIAA0101* gene expression profiles after NCT (SS), respectively. Blue lines, underexpression; red lines, overexpression. (e-f) OS curves considering *NUSAP1* and *PCLAF*/*KIAA0101* expression profiles from the Kaplan–Meier plotter website (https://kmplot.com), respectively. Black lines, underexpression; red lines, overexpression.

Patients were followed up for 46.5 months on average (SD = 20.34; range = 5.1–79.2 months). Supplementary Figure S6 shows that HER2+ patients have better overall survival, although significance levels were not reached (*p* − value = 0.07). *NUSAP1* and *PCLAF* expression patterns were compared against tumor relapse for disease-free survival and death due to BC for OS. Regarding DFS, the number of relapses was significantly higher in patients with overexpression of *NUSAP1* in the SS (38%, log-rank Mantel-Cox test, *χ*^2^ = 4.67, *p* − value = 0.03) (Figure 5(a)). Likewise, higher levels of *NUSAP1* gene expression in the SS were also associated with decreased OS, with a reduction from 84% to 50% (log-rank Mantel-Cox test), *χ*^2^ = 5.198, *p* − value = 0.02) (Figure 5(c)). Similarly, *PCLAF* overexpression negatively affected OS, with a reduction from 80% to 71% (log-rank Mantel-Cox test), *χ*^2^ = 0.40, *p* − value = 0.53 (Figures 5(b) and 5(d)). Comparisons of gene expression patterns from BS failed to classify responders and no responders. OS results were replicated by analyzing public data on 1402 patients from the Kaplan–Meier Plotter website (https://kmplot.com) [40, 41]. Low levels of *NUSAP1* and *PCLAF* were associated with greater OS (log-rank HR = 1.82, CI95% = 1.46–2.26, *p* − value = 6.2 × 10^−8^ and log-rank HR = 1.47, CI95% = 1.19–1.82, *p-*value = 0.0004, respectively; Figures 5(e) and 5(f), respectively).

## 4. Discussion

Omics technologies, global gene expression analyses, in particular, have had a significant impact on the understanding of BC biology, the classification of pathologic subtypes, the design of predictive algorithms, and, most importantly, the discovery and implementation of new and more effective therapies to control this disease [42]. All these advances have positioned BC as one of the archetypal entities in precision medicine. Improvements in the selection of therapies based on the different molecular subtypes of BC have yielded higher DFS and prolonged OS. However, a high proportion of patients who do not fully respond to the assigned therapy has been observed after a particular treatment time. Therefore, choosing the appropriate therapeutic regimen at the beginning of treatment is crucial. Indeed, defining the most appropriate therapies beyond the first line is challenging, especially in pretreated patients [43]. Consequently, analysis of the molecular response to NCT may offer an opportunity to define prognoses and alternative therapies in patients with a BC diagnosis [12].

In this work, we studied gene expression profiles obtained through unsupervised cluster analysis of BS and SS tissues in patients with pCR and non-pCR after NCT. After evaluating the functional enrichment analysis of the different transcriptional signatures, the contrast pCR vs. non-pCR in SS tissues was the most attractive based on a hypothesis-driven approach to identify potential biomarkers associated with clinical outcomes. This analysis unveiled 30 overexpressed and 13 underexpressed genes in treated tumors (Supplementary File S3a). Seven of these genes enriched the regulation of the mitotic cell cycle (*AURKA, CCDC8, CCNB1, FHL1, NUSAP1*, *RRM2,* and *UBE2C*). Interestingly, *CCNB1, NUSAP1, RRM2,* and *UBE2C*, including *PCLAF* and *UBE2T,* are part of a transcriptional signature identified in BC from the Middle East young women [44]. Moreover, *CCNB1*, *RRM2*, and *UBE2C* are included in the PAM50 signature for the molecular classification of BC lesions [45]. However, as far as we know, there are no reports of a genetic signature predicting BC response after NCT.

Some studies evaluating gene expression profiles and their association with a pCR have been already reported. For example, in Kolacinska's study in 2012, they analyzed biopsies from 42 patients before NCT (anthracyclines and taxanes) and identified seven differentially expressed genes (*BAX, CYP2D6, ERCC1, FOXC1, IRF1, MAP2*, and *MKI67*) in patients with pCR. We should note that this study performed target gene analysis rather than global expression analysis. The authors selected 23 genes according to their inclusion criteria and did not include prognostic value data or in-depth analysis of enriched signaling pathways in the comparisons [45].

On the other hand, an 80-Gene Molecular Subtyping Profile (BluePrint) was evaluated as a predictor of response to chemotherapy in 133 patients treated with neoadjuvant chemotherapy (anthracyclines and taxanes). The results showed that the majority of patients with pCR were basal-type or Her2-enriched breast tumors. Of the 80 genes that make up BluePrint, 48 have coincidences with those described in the PAM50 gene set, in addition, Luminal-type tumors show gene enrichment in the estrogen receptor pathway [46]. In addition to these clinical approaches, computational reanalysis studies have been also carried out, such as the work reported by Zhao in 2020 where the response to NCT in patients, mostly with TNBC, is evaluated. In this work, response probability scores (RPS) were calculated to predict response to chemotherapy for TNBC and whose accuracy is higher than other previously reported signatures. These results are similar to those reported for ER-positive tumors using MammaPrint and Oncotype DX and reflect the activities of pathways, including cell cycle pathways, related to the immune system and ECM [46].

We selected four genes for RT-qPCR validation analyses based on the differential gene expression results in the microarray and the functional enrichment analysis using these DEGs identified in SS biopsies. Two chosen genes were overexpressed (*MME* and *DST*) and two more were underexpressed (*NUSAP1* and *PCLAF*). These validation studies corroborated the expression patterns observed in the microarray analyses. Interestingly, the gene expression levels of *NUSAP1* and *PCLAF* were more discriminating in the RT-qPCR analyses, so they were chosen to perform the DSF and OS studies (Supplementary Figure S3).

DSF and OS studies based on expression levels in SS demonstrated that low *NUSAP1* expression was associated with better DFS. Similarly, *NUSAP1* and *PCLAF* underexpression were associated with increased OS (Figures 5(a)–5(f)).

The most important observation of this study is that the pCR achieved with the NCT regimens (cyclophosphamide/doxorubicin or cyclophosphamide/epirubicin) is associated with a significant decrease in the gene expression levels of *NUSAP1* and *PCLAF*. The association between clinical outcomes and transcriptional profile is consistent with the fact that the expression of these genes is involved in mitosis and DNA replication, respectively (Figure 2), fundamental processes involved in cancer progression, as will be discussed later. As reported, this clinical response presupposes better DFS and OS [11]. Furthermore, higher expression levels of these same genes in the tumor biopsy before treatment (BS) were associated with poorer survival, indicating that these genes are potential predictors of survival in diagnostic biopsies.

Our study suggests that the HER2+ subtype responds favorably to NCT (*p* − value=0.02) and that the luminal B subtype responds poorly, with no observed significant difference. Gene expression patterns of *NUSAP1* and *PCLAF* in different molecular subtypes of BC after NCT showed that *NUSAP1* was overexpressed in luminal B tumors compared to luminal A, HER2+, and triple-negative subtypes (Figure 4 and Supplementary Figure S4). Colak et al. reported overexpression of *NUSAP1* and *PCLAF* in ductal in situ and invasive ductal carcinoma compared to normal age-matched controls [44]. Tumor-infiltrating lymphocytes have been reported to modulate the NCT response in breast cancer [47]. Furthermore, in the same study, no correlations were observed between TIL counts and gene expression (*NUSAP1* and *PCLAF*) in BS tissues from patients with and without PCR.

The protein PCNA-associated factor encoded by *PCLAF* binds the PCNA protein, acts as a regulator of the number of centrosomes, and is involved in DNA repair during DNA replication [15]. Overexpressed *PCLAF* has also been associated with decreased survival in BC patients [15] but not the pathologic response to NCT. Similarly, *NUSAP1* gene expression levels showed a remarkable inverse correlation with survival (Figures 5(a), 5(c), and 5(e)). This gene encodes for nucleolar and spindle-associated protein 1, which binds to chromatin and microtubules and is critical for the cytokinesis spindle assembly during mitosis [16]. *NUSAP1* overexpression has been reported in bladder, cervical, colon, liver, lung, prostate, kidney, and breast cancers, glioblastoma, and oral squamous cell carcinoma [48–52]; multiple studies have correlated its overexpression with poor prognosis [15, 49, 50, 53–57]. Zhang et al. demonstrated that the downregulation of *NUSAP1* suppressed proliferation, migration, and invasion of MCF-7 cells by disturbing the regulation of *CDK1* and *DLGAP5* and increasing susceptibility to epirubicin [50]. Our findings are like those of Qiu et al. They reported higher *NUSAP1* expression in tumors than in adjacent healthy tissue and an inverse correlation between *NUSAP1* expression and OS in BC patients. These findings were corroborated in a BALB/c-nu mouse model in which they determined the involvement of *NUSAP1* in tumor proliferation, migration, and invasion [18]. Finally, *NUSAP1* has been proposed as a carcinogenic element whose overexpression would help tumor progression in triple-negative BC cells, participating in the epithelial-mesenchymal transition and the Wnt/*β*-catenin pathways [17].

Our findings, together with those previously reported, indicate that these two genes may be prognostic genetic markers in BC but, at the same time, potential therapeutic targets. The proteins encoded by *NUSAP1* and *PCLAF* are involved in BCRA1-mediated DNA repair. NUSAP1 increases *BRCA1* expression [58], whereas *PCLAF* regulates the number of centrosomes by interacting with BRCA1 [15]. Since the biological roles of the *NUSAP1* and *PCLAF* involve cell cycle pathways, patients with elevated transcription levels of these genes may benefit from chemotherapeutic drugs interfering with BRCA1, such as platinum derivatives. *NUSAP1* overexpression could also be treated with galiellalactone, a fungal metabolite with antitumor and anti-inflammatory properties. Galiellalactone downregulates *NUSAP1* in DU 145 cells by targeting the NF-*κ*B and STAT3 pathways, inducing cell cycle arrest [59]. Another option to target *NUSAP1* overexpression is the antitumor compound isopicrinine, isolated from *Rhazya stricta*, an inhibitor of the microtubule assembly [60].

Finally, decreased expression of *NUSAP1* seems to sensitize osteosarcoma cells to paclitaxel, as *NUSAP1* interacts with the RanBP2-RanGAP1-UBC9 SUMO E3 ligase complex, allowing for accurate chromosomal segregation [61]. In addition, *NUSAP1* knockdown has been observed to potentiate paclitaxel-induced apoptosis in oral squamous cell carcinoma [62].

Our studies show significant results of the downregulation of *NUSAP1* and *PCLAF* and overexpression of *MME* and *DST* in SS, predicting pCR. BS data do not reach significance, but this correlation is also registered. On the contrary, the data suggest that overexpression of *NUSAP1* and *PCLAF* are associated with decreased DFS. This information could be useful to implement second-line treatment or more aggressive regimens in nonresponders.

It is essential to highlight some limitations of this study. The first is the small sample size; however, the NCT schemes and sampling were standardized for most study participants. The selection also has an overrepresentation of triple-negative BC because the NCT program prioritizes patients with this tumor subtype.

## 5. Conclusions

Downregulation of *NUSAP1* and *PCLAF* in SS after NCT was associated with favorable therapeutic response and prognosis in BC. These two genes represent potential biomarkers for personalized therapies for patients who do not respond adequately to NCT.

## Figures and Tables

**Table 1:** Clinical characteristics of patients included in the study.

	All patients (*n* = 39)		pCR (*n* = 8) (20.5%)		Non-pCR (*n* = 31) (71.5%)		*p* − value
Age at diagnosis (years)	48	26 to 63	47	38 to 57	48	26 to 63	0.73

BMI (body mass index, kg/m^2^)	28.20	20.80 to 39.70	28.4	20.80 to 39.70	28.20	24.80 to 33.10	0.88
<25	8	20.51%	1	12.50%	7	22.58%	
>25	28	71.80%	6	75.00%	22	70.97%	
No data	3	7.69%	1	12.50%	2	6.45%	

Menopause status						
Pre	21	53.85%	5	62.50%	16	51.61%	0.88
Post	18	46.15%	3	37.50%	15	48.39%	

Family history						
Yes	19	48.72%	3	37.50%	16	51.61%	0.75
No	20	51.28%	5	62.50%	15	48.39%	

Diabetes mellitus						
Yes	2	5.13%	0	0.00%	2	6.45%	0.87
No	37	94.87%	8	100.00%	29	93.55%	
Number of children			3.6		3.2		0.44
Nulliparous	4	10.26%	0	0.00%	4	12.90%	0.22
1 or 2	12	30.77%	2	25.00%	10	32.26%	
>3	23	58.97%	6	75.00%	17	54.84%	

Lactation						
Yes	16	41.03%	3	37.50%	13	41.94%	0.97
No	11	28.20%	2	25.00%	9	29.03%	
No data	12	30.77%	3	37.50%	9	29.03%	

Smoking						
Yes	5	12.82%	2	25.0%	3	9.68%	0.25
No	34	87.18%	6	75.0%	28	90.32%	

Clinical stage						
I	1	2.56%	1	12.50%	0	0.00%	0.82
II	19	48.72%	2	25.00%	17	54.84%	
III	19	48.72%	5	62.50%	14	45.16%	

TNM classification						
T1	1	2.56%	1	12.50%	0	0.00%	0.93
T2	18	46.15%	1	12.50%	17	54.84%	
T3	11	28.21%	4	50.00%	7	22.58%	
T4	9	23.08%	2	25.00%	7	22.58%	
N0	7	17.95%	2	25.00%	5	16.13%	
N1	23	58.97%	4	50.00%	19	61.29%	
N2	9	23.08%	2	25.00%	7	22.58%	
M0	39	100.00%	8	100.0%	31	100.00%	

IHC markers						
ER+	16	41.03%	2	25.00%	14	45.16%	0.30
ER-	23	58.97%	6	75.00%	17	54.84%	

PR +	17	43.59%	2	25.00%	15	48.39%	0.23
PR-	22	56.41%	6	75.00%	16	51.61%	

HER2+	7	17.95%	5	62.50%	2	6.45%	0.002
HER2 -	32	82.05%	3	37.50%	29	93.55%	

ki67	15.40	5 to 70	17.14	5 to 50	14.92	2 to 70	0.76

Molecular subtype						
Luminal A	10	25.64%	1	12.50%	9	29.03%	0.51
Luminal B	6	15.39%	0	0.00%	6	19.36%	
HER2+	7	17.95%	5	62.50%	2	6.45%	
Triple-negative	16	41.02%	2	25.00%	14	45.16%	

*NUSAP1* (BS)^a^						
Overexpressed	17	43.59%	2	25.00%	15	48.39%	0.43
Underexpressed	22	56.41%	6	75.00%	16	51.61%	

*PCLAF* (BS)^a^						
Overexpressed	18	46.15%	4	50.00%	14	45.16%	>0.99
Underexpressed	21	53.85%	4	50.00%	17	54.84%	

*NUSAP1* (SS)^a^						
Overexpressed	12	30.77%	0	0.00%	12	38.71%	0.04
Underexpressed	27	69.23%	8	100.00%	19	61.29%	

*PCLAF* (SS)^a^						
Overexpressed	28	71.80%	1	12.50%	29	93.55%	0.0001
Underexpressed	11	28.20%	7	87.50%	2	6.45%	

a *NUSAP1* or PCLAF were identified as overexpressed or underexpressed based on the contrast pCR vs. non-pCR in BS or SS samples as specified.

**Table 2:** *NUSAP1* and *PCLAF* and response to treatment.

	*NUSAP1*	*PCLAF*
OR	0.00	0.01
CI95%	0.000 to 0.991	0.0009 to 0.1383
*p*-value	0.0417	0.0001
Sensitivity	0.00	0.13
CI95%	0.000 to 0.324	0.006412 to 0.4709
Specificity	0.61	0.06452
CI95%	0.438 to 0.763	0.01146 to 0.2072
Positive predictive value	0.00	0.03
CI95%	0.000 to 0.243	0.001710 to 0.1667
Negative predictive value	0.70	0.22
CI95%	0.515 to 0.842	0.03948 to 0.5474
Likelihood ratio	0.00	0.1336

## Data Availability

The dataset generated and analyzed during the current study can be available from the corresponding author upon reasonable request.

## Supplementary Materials

Supplementary Materials
*Supplementary Figure S1.* Heatmap of pCR samples: SS (*n* = 16) vs. BS (*n* = 16). In the top row, SS samples are denoted by the blue header, and the red title indicates BS samples. The heatmap shows one sample for each column and one gene or probe for each horizontal line. The color indicates gene expression value intensities, where the pink-red gradient represents overexpression, and the light blue–dark blue gradient represents underexpression. *SupplementaryFigure S2.* Heatmap of non-pCR samples: SS (*n* = 31) vs. BS (*n* = 31). In the top row, BS samples are denoted by the blue header, and the red title indicates SS samples. The heatmap shows one sample for each column and one gene or probe for each horizontal line. The color indicates gene expression value intensities, where the pink-red gradient represents overexpression, and the light blue–dark blue gradient represents underexpression. *Supplementary Figure S3.* Box plots showing microarray selected genes validation by RT-qPCR (*NUSAP1*, *PCLAF1*, *DST*, and *MME*). a and b represent expression levels of *NUSAP1* and *PCLAF1*, respectively. c and d represent the expression of *DST* and *MME*, respectively. An unpaired *t*-test with Welch's correction was used for comparisons. *Supplementary Figure S4.* Expression levels of *NUSAP1* according to the molecular subtype after NCT (SS). LA, luminal A; LB, luminal B; TN = triple negative. One-way ANOVA and the Holm–Sidak multiple comparisons test were used for comparisons. *Supplementary Figure S5*. Microscopic evaluation of tumor-infiltrating lymphocytes (TILs). (a) Low TILs, 10×. Fibrous stroma is observed between the tumor cells, with little lymphoplasmacytic infiltration at 5%. (b) Moderate TILs, 10×. Moderate lymphoplasmacytic infiltrate is seen in the tumoral stroma at 30%. (c) High TILs, 10×. A dense lymphoplasmacytic infiltrate was observed in the stroma between the neoplastic cells in the upper left area at 80%. *Supplementary Figure S6*. Overall survival according to the molecular subtype after NCT only in surgical samples. LA = luminal A, LB = luminal B, TN = triple negative. Log-rank (Mantel-Cox) test was used for comparisons. *Supplementary Figure S7.* Graphic abstract: Gene expression profiles based on microarrays were carried out in Breast Cancer (BC) tumor samples from Biopsy (BS) at the diagnostic time and from Surgery (SS) after neoadjuvant chemotherapy treatment (NCT) with cyclophosphamide-doxorubicin/epirubicin, to define tumor molecular adaptations to chemotherapy in patients who showed pathologic complete response (pCR) or therapeutic failure (non-pCR) after NCT. A signature of 43 differentially expressed genes discriminated pCR from non-pCR patients (|fold change >2|, false discovery rate <0.05) only in biopsies taken from surgery. Based on unsupervised clustering of gene expression, together with functional enrichment analyses of differentially expressed genes, we selected *NUSAP1*, *PCLAF*. We also analyze the correlation between *NUSAP1* and *PCLAF* against disease-free survival (DFS) and overall survival (OS). Patients achieving pCR showed downregulation of *NUSAP1* and *PCLAF* in tumor tissues and increased DFS and OS, while overexpression of these genes correlated with poor therapeutic response and OS. These genes are involved in the regulation of mitotic division. Conclusions: the downregulation of *NUSAP1* and *PCLAF* after NCT is associated with the tumor response to chemotherapy and patient survival. *Supplementary File S1*. (a) List of fourteen differentially expressed genes (DEGs) in the comparison of pCR-SS vs. pCR-BS. (b) Functional enrichment analysis of this contrast using 14 DEGs. *Supplementary File S2*. (a) List of four differentially expressed genes in the contrast non-pCR-SS vs. non-pCR-BS. (b) Functional enrichment analysis of this comparison using 4 DEGs. *Supplementary File S3*. (a) List of forty-three differentially expressed genes in the comparison of pCR-SS vs. non-pCR-SS. (b) Functional enrichment analysis of this contrast using 43 DEGs. (c) Enriched bio-functions defined by the Ingenuity Knowledge Base. (d) Enriched canonical pathways defined by the Ingenuity Knowledge Base.
