# Supplementary material for: NUSAP1 and PCLAF (KIA0101) Downregulation by Neoadjuvant Therapy is Associated with Better Therapeutic Outcomes and Survival in Breast Cancer
Source: J Oncol. 2022 Nov 28;2022:6001947. doi: 10.1155/2022/6001947 (PMC9722309; doi:10.1155/2022/6001947)
Supplement: Supplementary Materials — Supplementary Figure S1. Heatmap of pCR samples: SS (n = 16) vs. BS (n = 16). In the top row, SS samples are denoted by the blue header, and the red title indicates BS samples. The heatmap shows one sample for each column and one gene or probe for each horizontal line. The color indicates gene expression value intensities, where the pink-red gradient represents overexpression, and the light blue–dark blue gradient represents underexpression. SupplementaryFigure S2. Heatmap of non-pCR samples: SS (n = 31) vs. BS (n = 31). In the top row, BS samples are denoted by the blue header, and the red title indicates SS samples. The heatmap shows one sample for each column and one gene or probe for each horizontal line. The color indicates gene expression value intensities, where the pink-red gradient represents overexpression, and the light blue–dark blue gradient represents underexpression. Supplementary Figure S3. Box plots showing microarray selected genes validation by RT-qPCR (NUSAP1, PCLAF1, DST, and MME). a and b represent expression levels of NUSAP1 and PCLAF1, respectively. c and d represent the expression of DST and MME, respectively. An unpaired t-test with Welch's correction was used for comparisons. Supplementary Figure S4. Expression levels of NUSAP1 according to the molecular subtype after NCT (SS). LA, luminal A; LB, luminal B; TN = triple negative. One-way ANOVA and the Holm–Sidak multiple comparisons test were used for comparisons. Supplementary Figure S5. Microscopic evaluation of tumor-infiltrating lymphocytes (TILs). (a) Low TILs, 10×. Fibrous stroma is observed between the tumor cells, with little lymphoplasmacytic infiltration at 5%. (b) Moderate TILs, 10×. Moderate lymphoplasmacytic infiltrate is seen in the tumoral stroma at 30%. (c) High TILs, 10×. A dense lymphoplasmacytic infiltrate was observed in the stroma between the neoplastic cells in the upper left area at 80%. Supplementary Figure S6. Overall survival according to the molecular subtype afte [file 6001947.f1.zip › Supplementary Figure S7 Graphic abstract.pptx]

## Slide 1
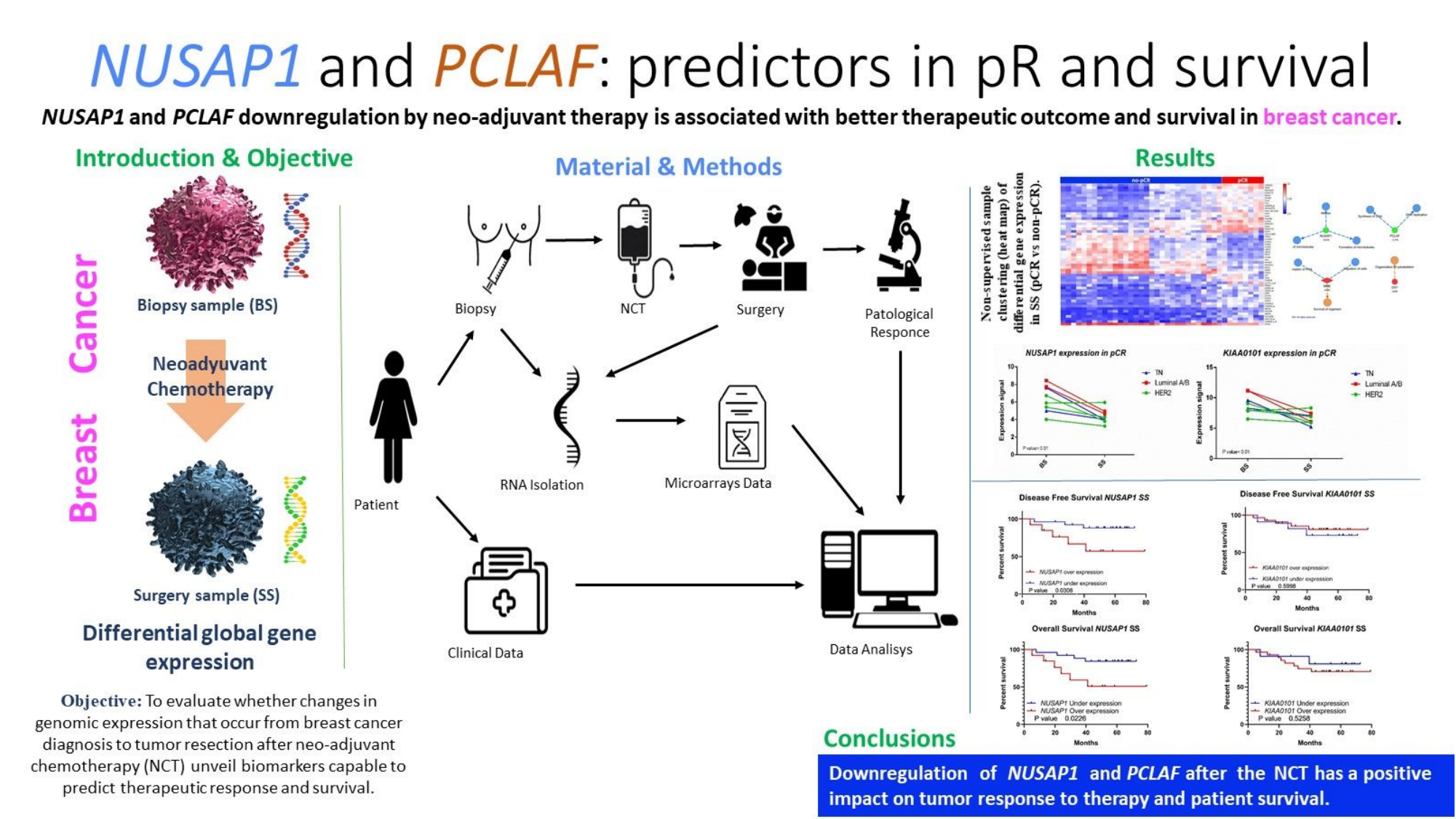

## Slide 2
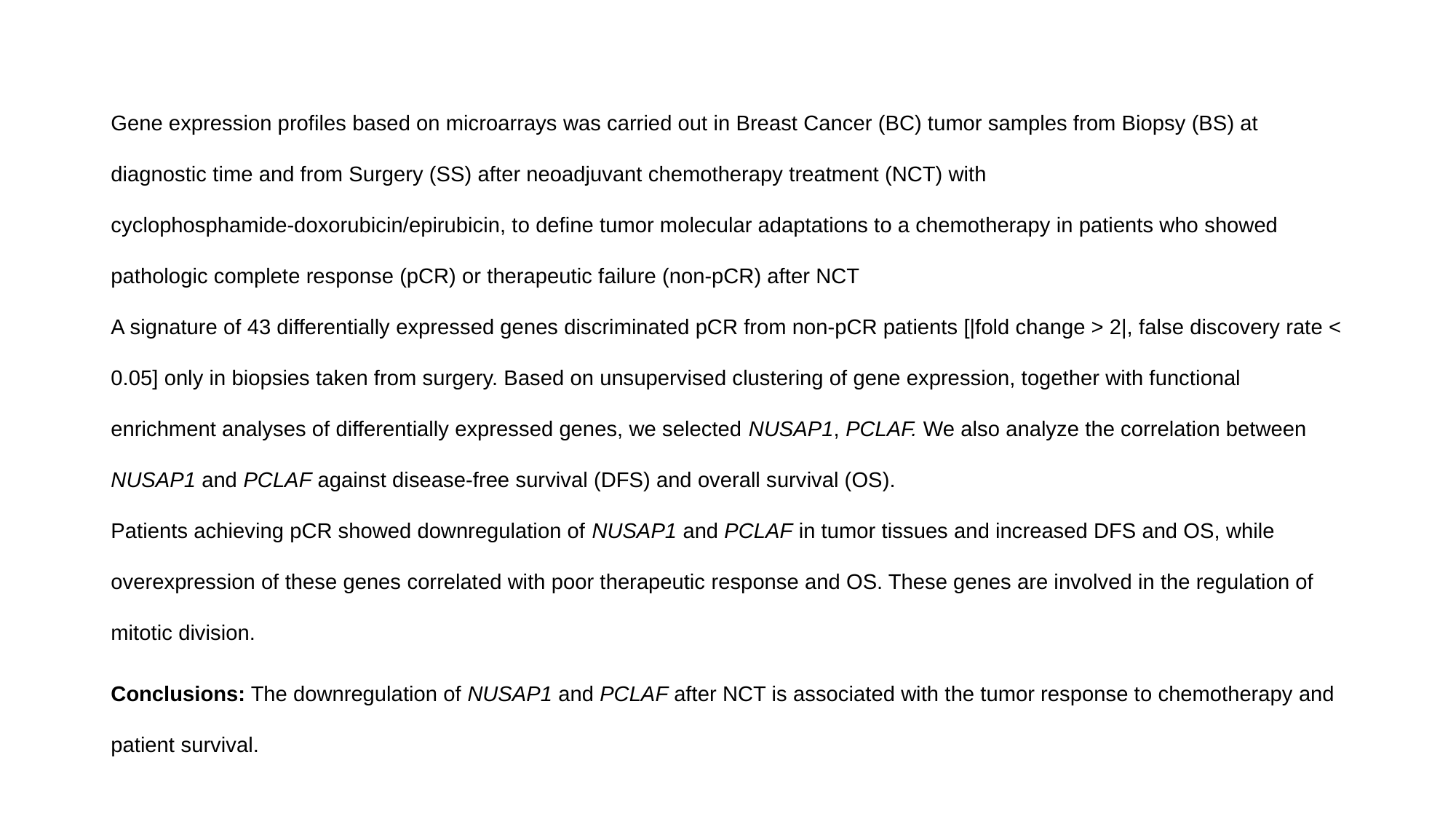

Gene expression profiles based on microarrays was carried out in Breast Cancer (BC) tumor samples from Biopsy (BS) at diagnostic time and from Surgery (SS) after neoadjuvant chemotherapy treatment (NCT) with cyclophosphamide-doxorubicin/epirubicin, to define tumor molecular adaptations to a chemotherapy in patients who showed pathologic complete response (pCR) or therapeutic failure (non-pCR) after NCT
A signature of 43 differentially expressed genes discriminated pCR from non-pCR patients [|fold change > 2|, false discovery rate < 0.05] only in biopsies taken from surgery. Based on unsupervised clustering of gene expression, together with functional enrichment analyses of differentially expressed genes, we selected NUSAP1, PCLAF. We also analyze the correlation between NUSAP1 and PCLAF against disease-free survival (DFS) and overall survival (OS).
Patients achieving pCR showed downregulation of NUSAP1 and PCLAF in tumor tissues and increased DFS and OS, while overexpression of these genes correlated with poor therapeutic response and OS. These genes are involved in the regulation of mitotic division.
Conclusions: The downregulation of NUSAP1 and PCLAF after NCT is associated with the tumor response to chemotherapy and patient survival.
