# Supplementary figures and images for: NUSAP1 and PCLAF (KIA0101) Downregulation by Neoadjuvant Therapy is Associated with Better Therapeutic Outcomes and Survival in Breast Cancer
Source: J Oncol. 2022 Nov 28;2022:6001947. doi: 10.1155/2022/6001947 (PMC9722309; doi:10.1155/2022/6001947)

## Slide 1
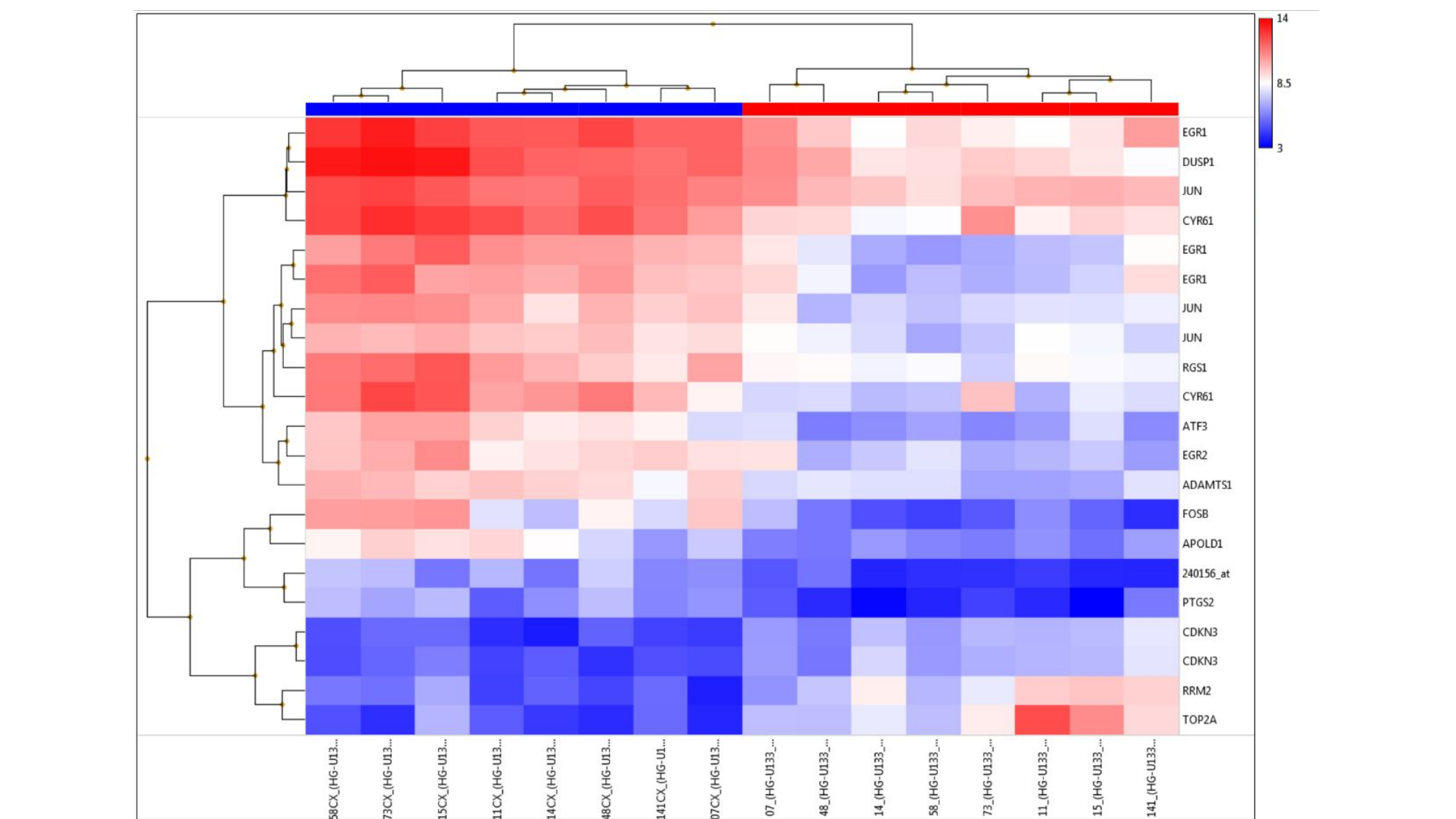

Supplement: Supplementary Materials — Supplementary Figure S1. Heatmap of pCR samples: SS (n = 16) vs. BS (n = 16). In the top row, SS samples are denoted by the blue header, and the red title indicates BS samples. The heatmap shows one sample for each column and one gene or probe for each horizontal line. The color indicates gene expression value intensities, where the pink-red gradient represents overexpression, and the light blue–dark blue gradient represents underexpression. SupplementaryFigure S2. Heatmap of non-pCR samples: SS (n = 31) vs. BS (n = 31). In the top row, BS samples are denoted by the blue header, and the red title indicates SS samples. The heatmap shows one sample for each column and one gene or probe for each horizontal line. The color indicates gene expression value intensities, where the pink-red gradient represents overexpression, and the light blue–dark blue gradient represents underexpression. Supplementary Figure S3. Box plots showing microarray selected genes validation by RT-qPCR (NUSAP1, PCLAF1, DST, and MME). a and b represent expression levels of NUSAP1 and PCLAF1, respectively. c and d represent the expression of DST and MME, respectively. An unpaired t-test with Welch's correction was used for comparisons. Supplementary Figure S4. Expression levels of NUSAP1 according to the molecular subtype after NCT (SS). LA, luminal A; LB, luminal B; TN = triple negative. One-way ANOVA and the Holm–Sidak multiple comparisons test were used for comparisons. Supplementary Figure S5. Microscopic evaluation of tumor-infiltrating lymphocytes (TILs). (a) Low TILs, 10×. Fibrous stroma is observed between the tumor cells, with little lymphoplasmacytic infiltration at 5%. (b) Moderate TILs, 10×. Moderate lymphoplasmacytic infiltrate is seen in the tumoral stroma at 30%. (c) High TILs, 10×. A dense lymphoplasmacytic infiltrate was observed in the stroma between the neoplastic cells in the upper left area at 80%. Supplementary Figure S6. Overall survival according to the molecular subtype afte [file 6001947.f1.zip › FIG S1.pptx]

## Slide 1
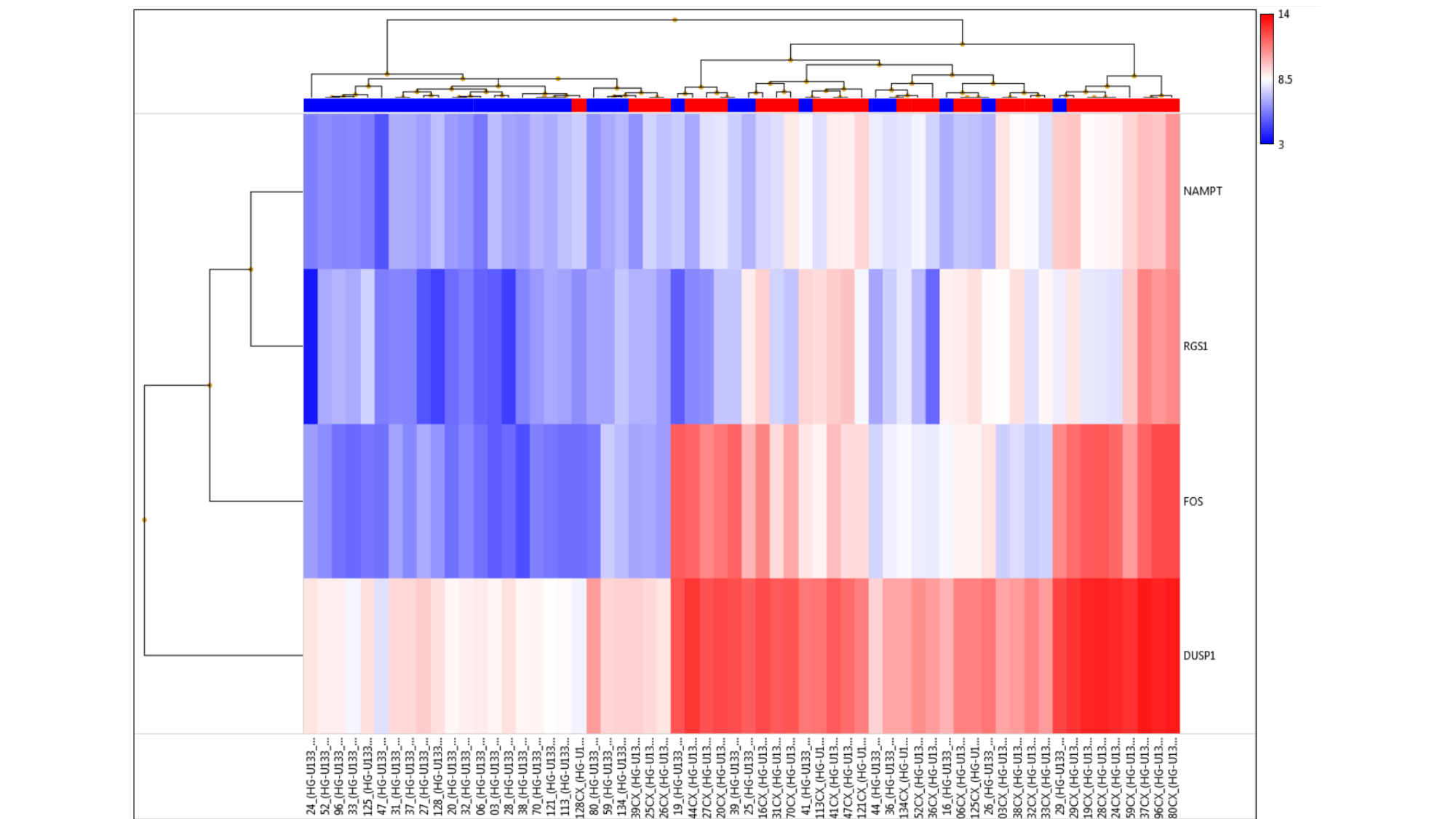

Supplement: Supplementary Materials — Supplementary Figure S1. Heatmap of pCR samples: SS (n = 16) vs. BS (n = 16). In the top row, SS samples are denoted by the blue header, and the red title indicates BS samples. The heatmap shows one sample for each column and one gene or probe for each horizontal line. The color indicates gene expression value intensities, where the pink-red gradient represents overexpression, and the light blue–dark blue gradient represents underexpression. SupplementaryFigure S2. Heatmap of non-pCR samples: SS (n = 31) vs. BS (n = 31). In the top row, BS samples are denoted by the blue header, and the red title indicates SS samples. The heatmap shows one sample for each column and one gene or probe for each horizontal line. The color indicates gene expression value intensities, where the pink-red gradient represents overexpression, and the light blue–dark blue gradient represents underexpression. Supplementary Figure S3. Box plots showing microarray selected genes validation by RT-qPCR (NUSAP1, PCLAF1, DST, and MME). a and b represent expression levels of NUSAP1 and PCLAF1, respectively. c and d represent the expression of DST and MME, respectively. An unpaired t-test with Welch's correction was used for comparisons. Supplementary Figure S4. Expression levels of NUSAP1 according to the molecular subtype after NCT (SS). LA, luminal A; LB, luminal B; TN = triple negative. One-way ANOVA and the Holm–Sidak multiple comparisons test were used for comparisons. Supplementary Figure S5. Microscopic evaluation of tumor-infiltrating lymphocytes (TILs). (a) Low TILs, 10×. Fibrous stroma is observed between the tumor cells, with little lymphoplasmacytic infiltration at 5%. (b) Moderate TILs, 10×. Moderate lymphoplasmacytic infiltrate is seen in the tumoral stroma at 30%. (c) High TILs, 10×. A dense lymphoplasmacytic infiltrate was observed in the stroma between the neoplastic cells in the upper left area at 80%. Supplementary Figure S6. Overall survival according to the molecular subtype afte [file 6001947.f1.zip › FIG S2.pptx]

## Slide 1
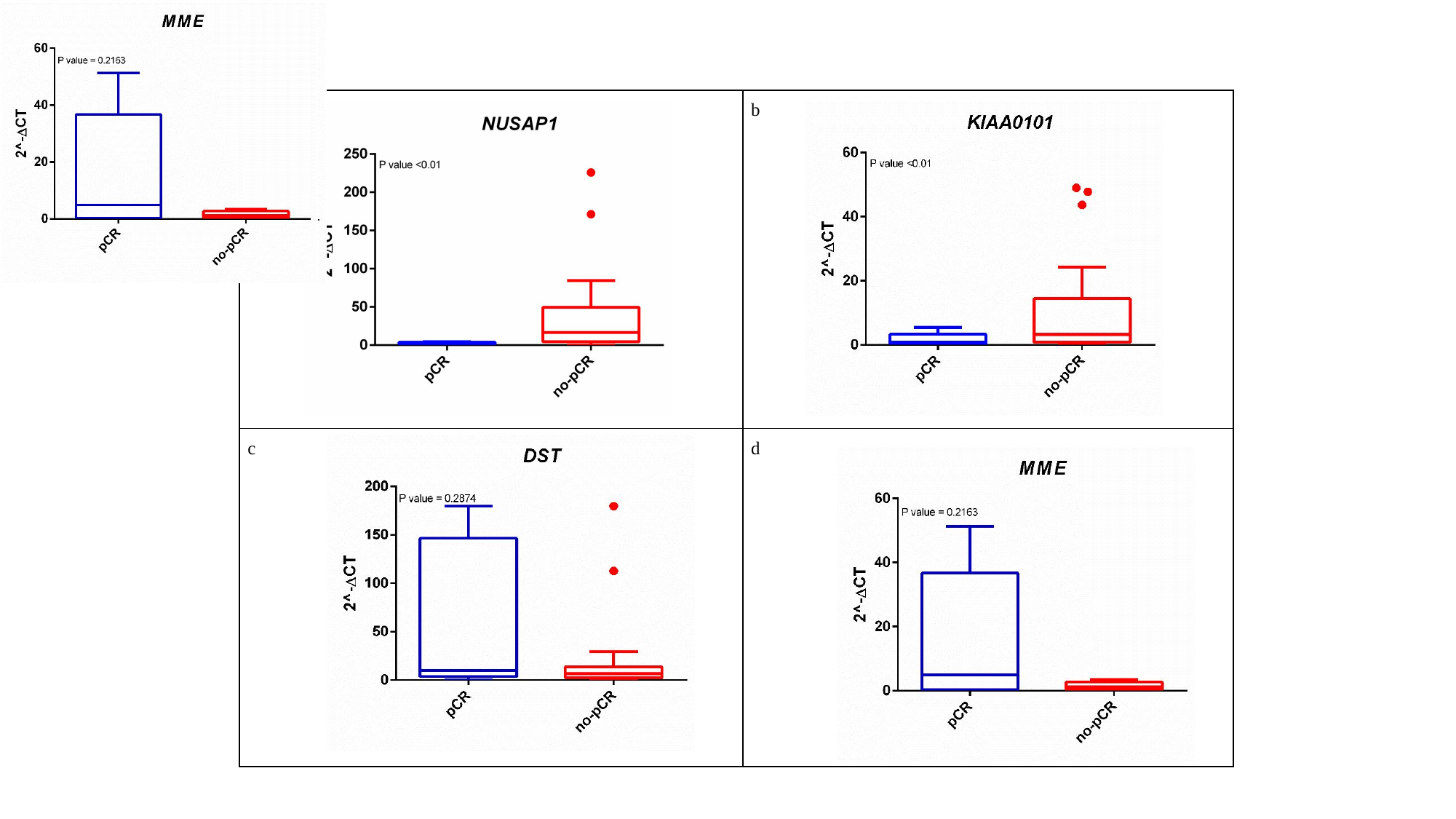

| a | b |
| --- | --- |
| c | d |

## Slide 2
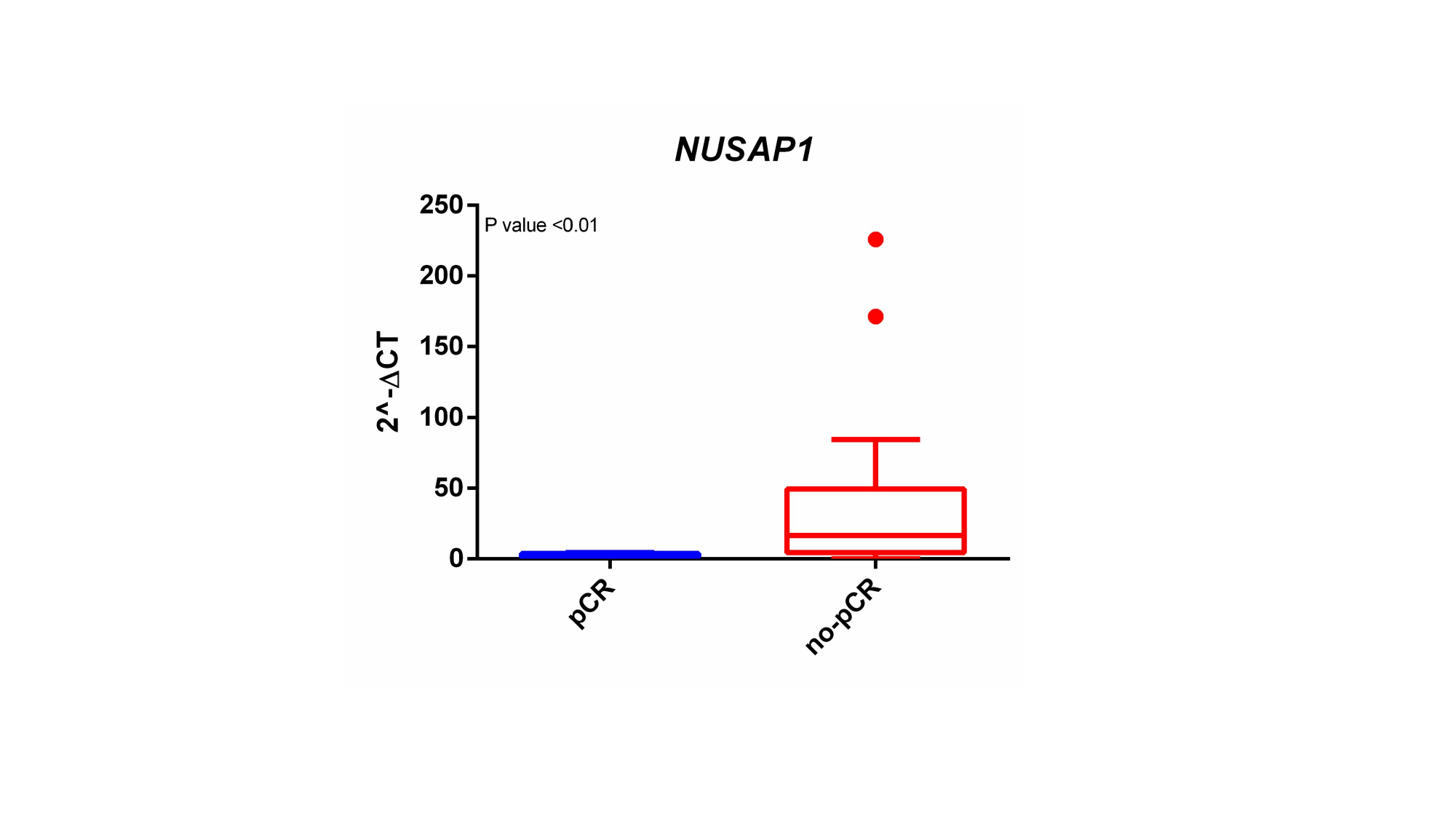

## Slide 3
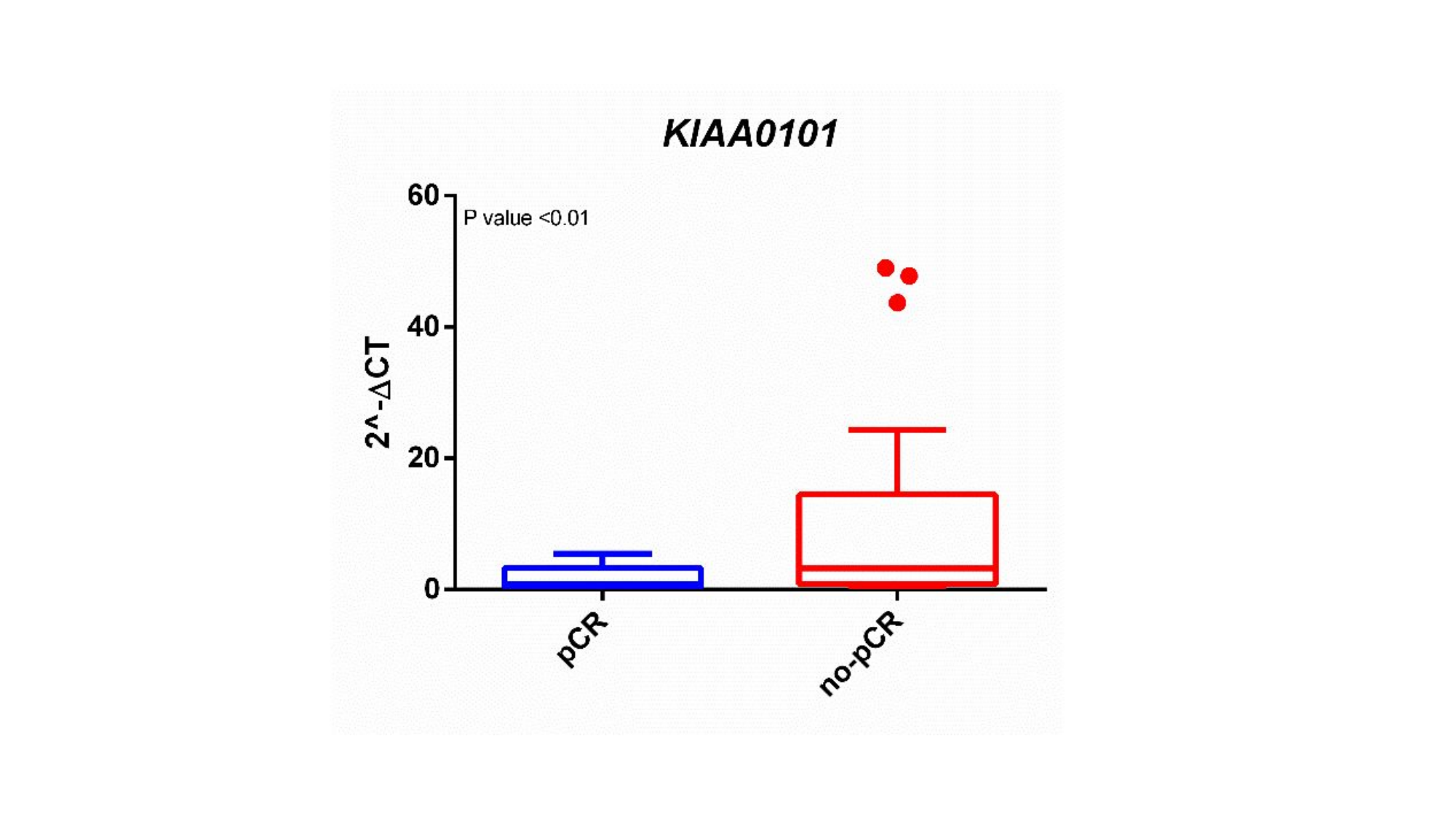

## Slide 4
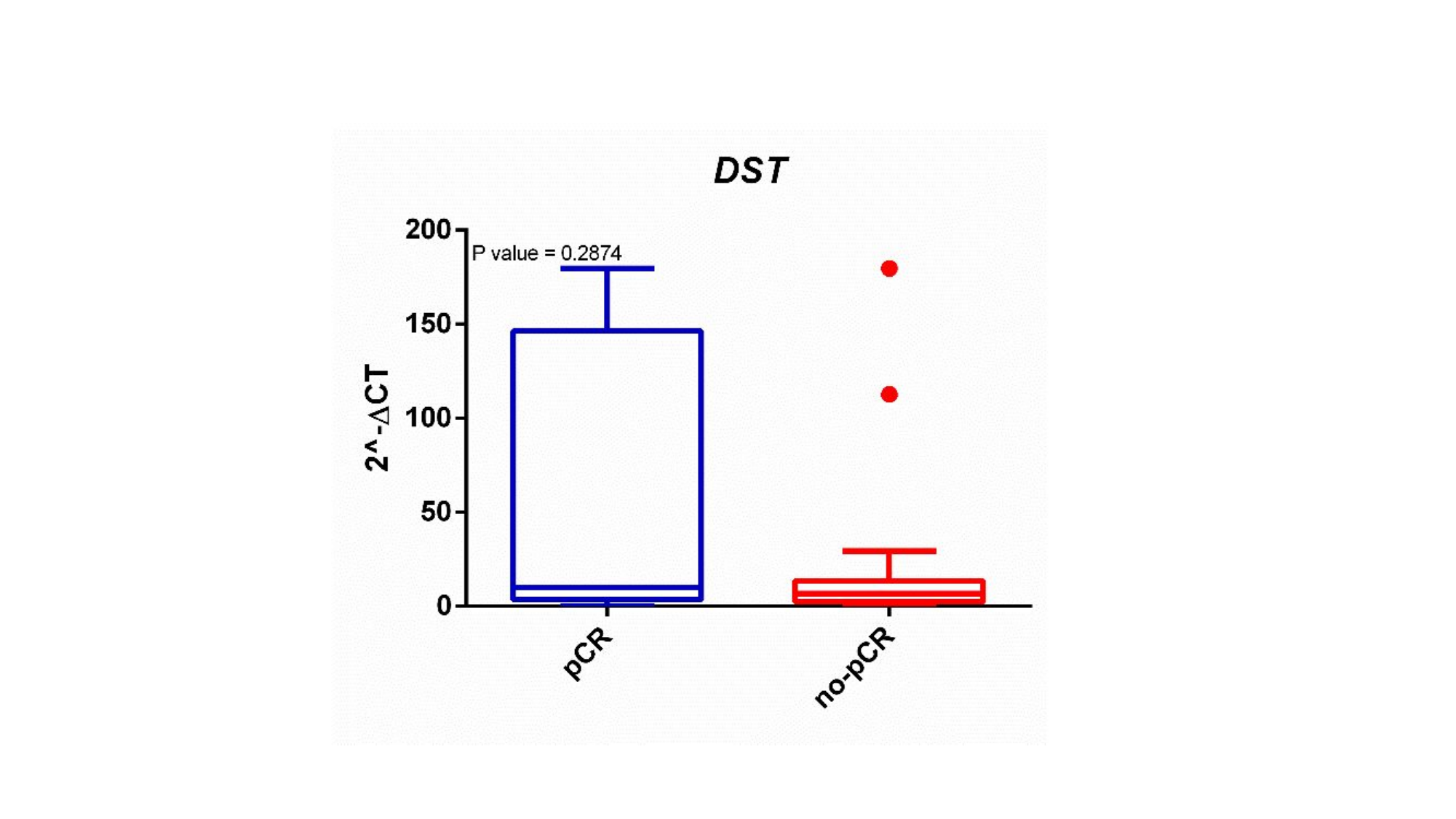

## Slide 5
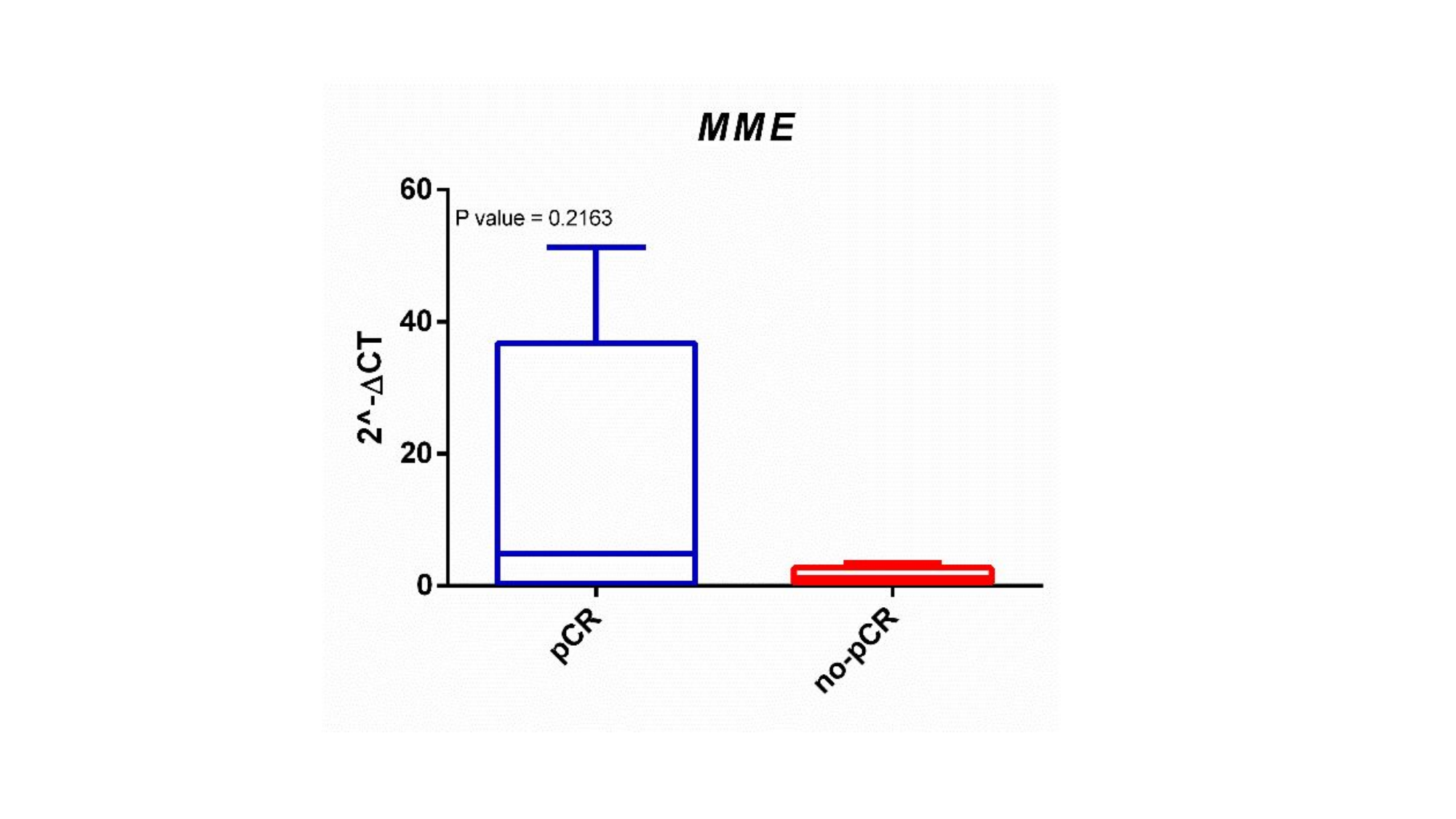

Supplement: Supplementary Materials — Supplementary Figure S1. Heatmap of pCR samples: SS (n = 16) vs. BS (n = 16). In the top row, SS samples are denoted by the blue header, and the red title indicates BS samples. The heatmap shows one sample for each column and one gene or probe for each horizontal line. The color indicates gene expression value intensities, where the pink-red gradient represents overexpression, and the light blue–dark blue gradient represents underexpression. SupplementaryFigure S2. Heatmap of non-pCR samples: SS (n = 31) vs. BS (n = 31). In the top row, BS samples are denoted by the blue header, and the red title indicates SS samples. The heatmap shows one sample for each column and one gene or probe for each horizontal line. The color indicates gene expression value intensities, where the pink-red gradient represents overexpression, and the light blue–dark blue gradient represents underexpression. Supplementary Figure S3. Box plots showing microarray selected genes validation by RT-qPCR (NUSAP1, PCLAF1, DST, and MME). a and b represent expression levels of NUSAP1 and PCLAF1, respectively. c and d represent the expression of DST and MME, respectively. An unpaired t-test with Welch's correction was used for comparisons. Supplementary Figure S4. Expression levels of NUSAP1 according to the molecular subtype after NCT (SS). LA, luminal A; LB, luminal B; TN = triple negative. One-way ANOVA and the Holm–Sidak multiple comparisons test were used for comparisons. Supplementary Figure S5. Microscopic evaluation of tumor-infiltrating lymphocytes (TILs). (a) Low TILs, 10×. Fibrous stroma is observed between the tumor cells, with little lymphoplasmacytic infiltration at 5%. (b) Moderate TILs, 10×. Moderate lymphoplasmacytic infiltrate is seen in the tumoral stroma at 30%. (c) High TILs, 10×. A dense lymphoplasmacytic infiltrate was observed in the stroma between the neoplastic cells in the upper left area at 80%. Supplementary Figure S6. Overall survival according to the molecular subtype afte [file 6001947.f1.zip › FIG S3.pptx]

## Slide 1
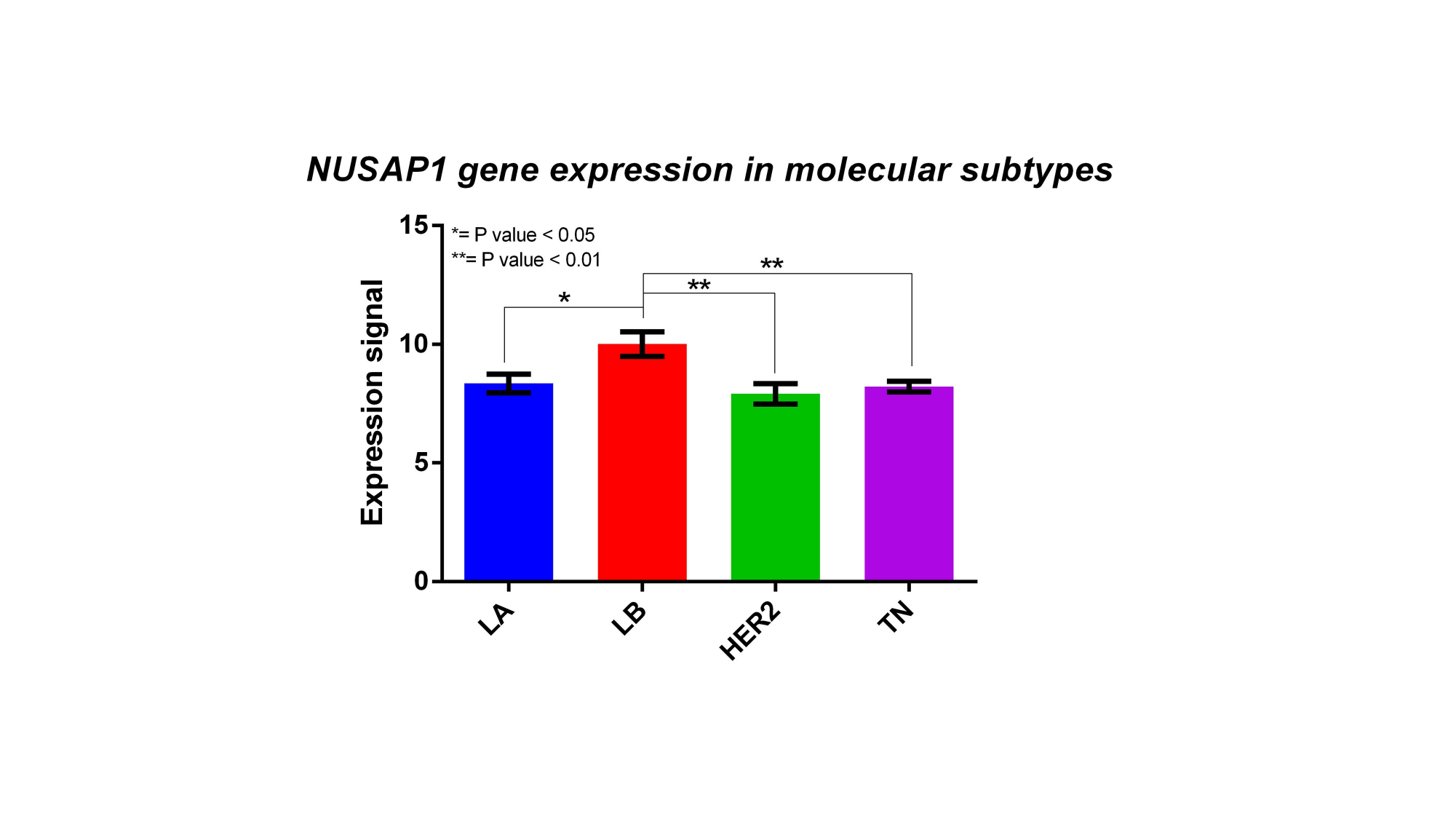

Supplement: Supplementary Materials — Supplementary Figure S1. Heatmap of pCR samples: SS (n = 16) vs. BS (n = 16). In the top row, SS samples are denoted by the blue header, and the red title indicates BS samples. The heatmap shows one sample for each column and one gene or probe for each horizontal line. The color indicates gene expression value intensities, where the pink-red gradient represents overexpression, and the light blue–dark blue gradient represents underexpression. SupplementaryFigure S2. Heatmap of non-pCR samples: SS (n = 31) vs. BS (n = 31). In the top row, BS samples are denoted by the blue header, and the red title indicates SS samples. The heatmap shows one sample for each column and one gene or probe for each horizontal line. The color indicates gene expression value intensities, where the pink-red gradient represents overexpression, and the light blue–dark blue gradient represents underexpression. Supplementary Figure S3. Box plots showing microarray selected genes validation by RT-qPCR (NUSAP1, PCLAF1, DST, and MME). a and b represent expression levels of NUSAP1 and PCLAF1, respectively. c and d represent the expression of DST and MME, respectively. An unpaired t-test with Welch's correction was used for comparisons. Supplementary Figure S4. Expression levels of NUSAP1 according to the molecular subtype after NCT (SS). LA, luminal A; LB, luminal B; TN = triple negative. One-way ANOVA and the Holm–Sidak multiple comparisons test were used for comparisons. Supplementary Figure S5. Microscopic evaluation of tumor-infiltrating lymphocytes (TILs). (a) Low TILs, 10×. Fibrous stroma is observed between the tumor cells, with little lymphoplasmacytic infiltration at 5%. (b) Moderate TILs, 10×. Moderate lymphoplasmacytic infiltrate is seen in the tumoral stroma at 30%. (c) High TILs, 10×. A dense lymphoplasmacytic infiltrate was observed in the stroma between the neoplastic cells in the upper left area at 80%. Supplementary Figure S6. Overall survival according to the molecular subtype afte [file 6001947.f1.zip › FIG S4.pptx]

## Slide 1
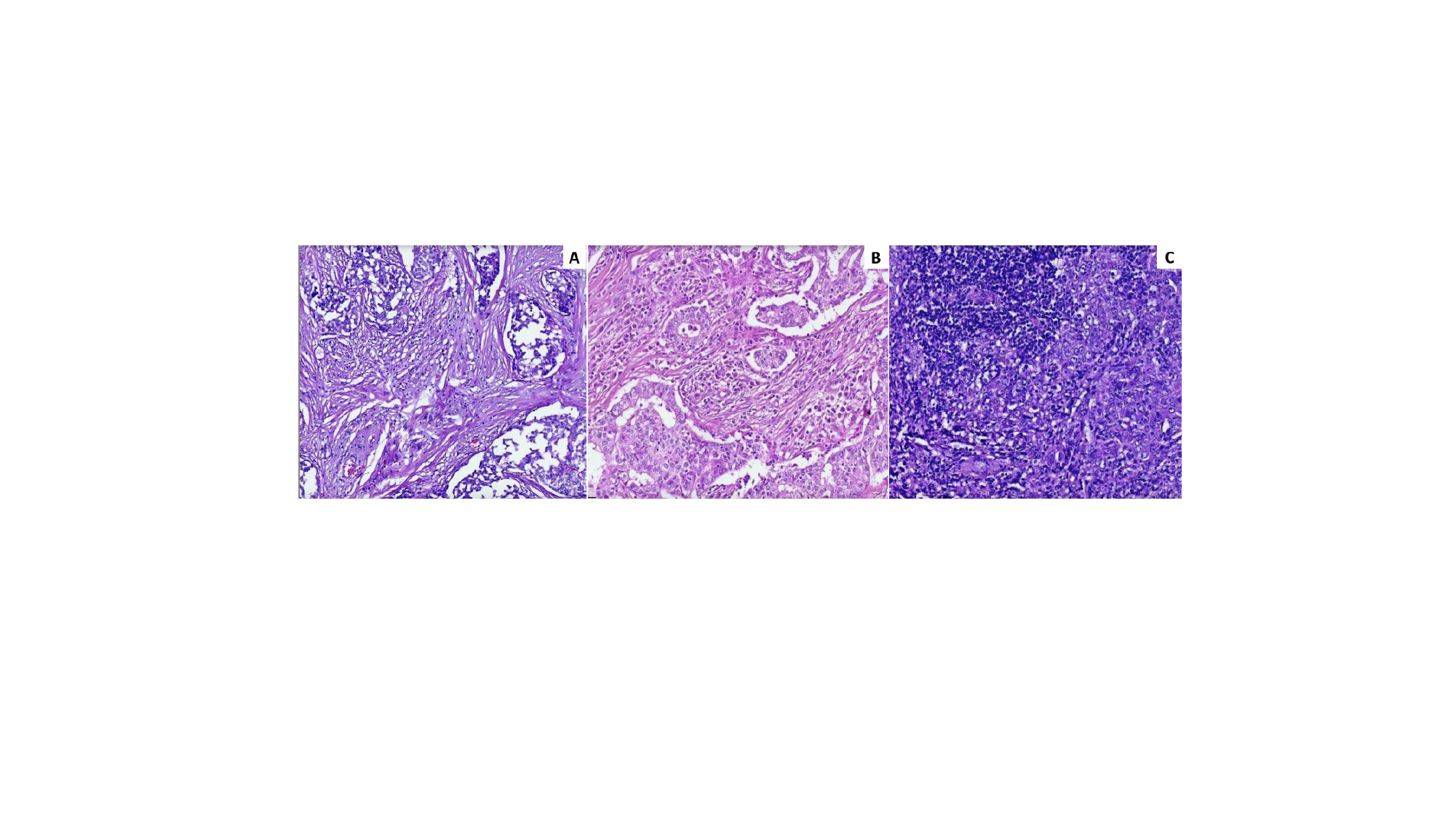

Supplement: Supplementary Materials — Supplementary Figure S1. Heatmap of pCR samples: SS (n = 16) vs. BS (n = 16). In the top row, SS samples are denoted by the blue header, and the red title indicates BS samples. The heatmap shows one sample for each column and one gene or probe for each horizontal line. The color indicates gene expression value intensities, where the pink-red gradient represents overexpression, and the light blue–dark blue gradient represents underexpression. SupplementaryFigure S2. Heatmap of non-pCR samples: SS (n = 31) vs. BS (n = 31). In the top row, BS samples are denoted by the blue header, and the red title indicates SS samples. The heatmap shows one sample for each column and one gene or probe for each horizontal line. The color indicates gene expression value intensities, where the pink-red gradient represents overexpression, and the light blue–dark blue gradient represents underexpression. Supplementary Figure S3. Box plots showing microarray selected genes validation by RT-qPCR (NUSAP1, PCLAF1, DST, and MME). a and b represent expression levels of NUSAP1 and PCLAF1, respectively. c and d represent the expression of DST and MME, respectively. An unpaired t-test with Welch's correction was used for comparisons. Supplementary Figure S4. Expression levels of NUSAP1 according to the molecular subtype after NCT (SS). LA, luminal A; LB, luminal B; TN = triple negative. One-way ANOVA and the Holm–Sidak multiple comparisons test were used for comparisons. Supplementary Figure S5. Microscopic evaluation of tumor-infiltrating lymphocytes (TILs). (a) Low TILs, 10×. Fibrous stroma is observed between the tumor cells, with little lymphoplasmacytic infiltration at 5%. (b) Moderate TILs, 10×. Moderate lymphoplasmacytic infiltrate is seen in the tumoral stroma at 30%. (c) High TILs, 10×. A dense lymphoplasmacytic infiltrate was observed in the stroma between the neoplastic cells in the upper left area at 80%. Supplementary Figure S6. Overall survival according to the molecular subtype afte [file 6001947.f1.zip › FIG S5.pptx]

## Slide 1
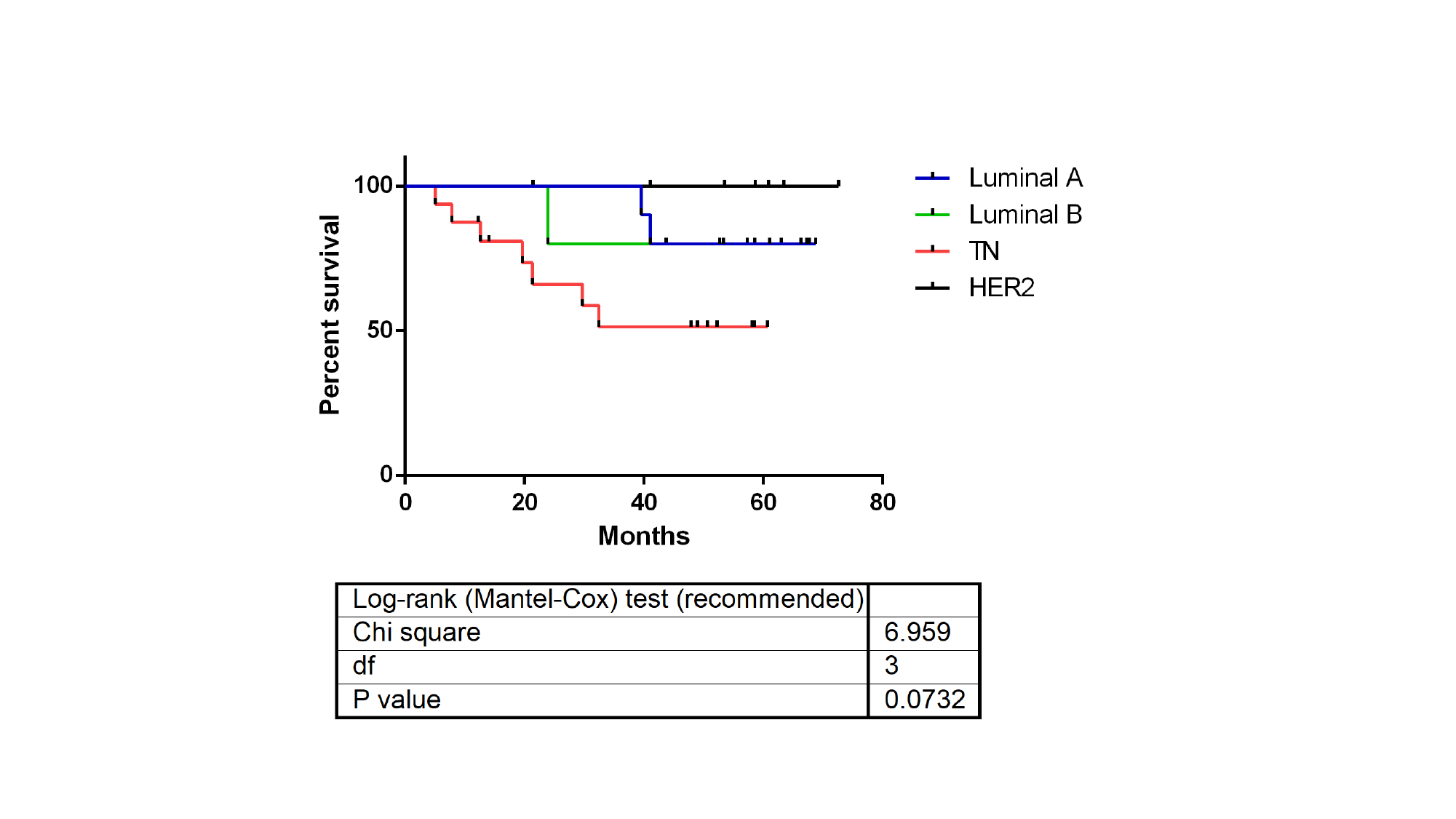

Supplement: Supplementary Materials — Supplementary Figure S1. Heatmap of pCR samples: SS (n = 16) vs. BS (n = 16). In the top row, SS samples are denoted by the blue header, and the red title indicates BS samples. The heatmap shows one sample for each column and one gene or probe for each horizontal line. The color indicates gene expression value intensities, where the pink-red gradient represents overexpression, and the light blue–dark blue gradient represents underexpression. SupplementaryFigure S2. Heatmap of non-pCR samples: SS (n = 31) vs. BS (n = 31). In the top row, BS samples are denoted by the blue header, and the red title indicates SS samples. The heatmap shows one sample for each column and one gene or probe for each horizontal line. The color indicates gene expression value intensities, where the pink-red gradient represents overexpression, and the light blue–dark blue gradient represents underexpression. Supplementary Figure S3. Box plots showing microarray selected genes validation by RT-qPCR (NUSAP1, PCLAF1, DST, and MME). a and b represent expression levels of NUSAP1 and PCLAF1, respectively. c and d represent the expression of DST and MME, respectively. An unpaired t-test with Welch's correction was used for comparisons. Supplementary Figure S4. Expression levels of NUSAP1 according to the molecular subtype after NCT (SS). LA, luminal A; LB, luminal B; TN = triple negative. One-way ANOVA and the Holm–Sidak multiple comparisons test were used for comparisons. Supplementary Figure S5. Microscopic evaluation of tumor-infiltrating lymphocytes (TILs). (a) Low TILs, 10×. Fibrous stroma is observed between the tumor cells, with little lymphoplasmacytic infiltration at 5%. (b) Moderate TILs, 10×. Moderate lymphoplasmacytic infiltrate is seen in the tumoral stroma at 30%. (c) High TILs, 10×. A dense lymphoplasmacytic infiltrate was observed in the stroma between the neoplastic cells in the upper left area at 80%. Supplementary Figure S6. Overall survival according to the molecular subtype afte [file 6001947.f1.zip › FIG S6.pptx]
